# Global and regional prevalence of osteoporosis in kidney transplant recipients: a systematic review and meta-analysis

**DOI:** 10.1007/s10238-025-01716-w

**Published:** 2025-06-20

**Authors:** Mobin Ghazaiean, Tahoora Mousavi, Mahmood Moosazadeh

**Affiliations:** 1 Student Research Committee, School of Medicine, Mazandaran University of Medical Sciences, Sari, Iran; 2 Gut and Liver Research Center, Non-communicable Disease Institute, Mazandaran University of Medical Sciences, Sari, Iran; 3 Molecular and Cell Biology Research Center, Hemoglobinopathy Institute, Mazandaran University of Medical Sciences, Sari, Iran; 4 Gastrointestinal Cancer Research Center, Non-Communicable Diseases Institute, Mazandaran University of Medical Sciences, Sari, Iran

**Keywords:** Osteoporosis, Prevalence, Kidney transplant recipients, Adult, Epidemiology

## Abstract

Kidney transplant recipients (KTRs) are at a heightened risk of low bone density and fractures. This study sought to determine the overall prevalence of osteoporosis in KTRs. Published observational studies and those with a baseline report on osteoporosis were evaluated in databases such as PubMed, Scopus, Science Direct, Web of Science, and EMBASE, in addition to Google Scholar from January 1, 2000, to January 1, 2024. The methodological quality of the included studies was rigorously assessed using the JBI Critical Appraisal checklist. Heterogeneity across primary study results was evaluated using the I-square index, while publication bias was scrutinized using Egger’s test and funnel plots. Statistical analyses were carried out using Stata software Ver. 11. After conducting searches across multiple databases, a total of 136 primary studies were ultimately included. The prevalence of osteoporosis in specific skeletal areas is as follows: 20% (95% CI 18–23, *n* = 67) in the lumbar region, 23% (95% CI 19–26, *n* = 53) in the femoral neck, 15% (95% CI 11–19, *n* = 20) in the total hip, 29% (95% CI 10–49, *n* = 5) in the forearm, and 34% (95% CI 28–40, *n* = 6) in the ultradistal radius. Based on sex-specific rate in the existing literature, the prevalence is reported as 21% (95% CI 15–27, *n* = 16) in men and 28% (95% CI 20–35, *n* = 16) in women. Our results for adult KTRs represent the high prevalence of osteoporosis which was highest in bone site rich in cortical content (e.g., ultradistal radius).

**Supplementary Information:**

The online version contains supplementary material available at 10.1007/s10238-025-01716-w.

## Introduction

Osteoporosis is a metabolic condition which characterized by a decrease in bone strength, leading to reduced bone mass, increased skeletal fragility, and deterioration of bone tissue structure. This condition particularly affects the spine, proximal femur, distal forearm, and proximal humerus [1]. The low bone mineral density (BMD) has sparked significant worry in nephrology, marked by weakened bone strength, which heightens the individual's susceptibility to fractures [2]. A study published in 2016 indicated that chronic kidney disease (CKD) is highly prevalent worldwide, with a steady estimated global prevalence ranging from 11 to 13%, primarily in CKD stage 3 [3]. According to research released in 2022, the worldwide prevalence of low BMD (T-score ≤ − 2.5) among patients with CKD was estimated to be 24.5% (95% CI 21.3–27.8%) [4]. Earlier research indicated that CKD and low BMD frequently occur together, with increased prevalence in later stages [5, 6]. In the last 20 years, considerable progress in kidney transplantation (KT) has produced notable enhancements, leading to frequent long-term survival for KTRs. KT focuses on restoring kidney function and the equilibrium of hormones that regulate minerals, along with the overall balance of mineral metabolites. Nevertheless, in spite of these measures, it is widely recognized that bone and mineral metabolism continue to be disturbed after transplantation and osteoporosis frequently arises after KT [7]. Identifying risk factors, which includes conventional risk factors, kidney-specific ones, fall risk, and frailty, should be considered in the pretransplant assessment [8]. Conventional risk factors that increase the likelihood of osteoporosis in patients comprise age, gender, low body mass index (BMI, ≤ 19 kg/m^2^), history of fragility fractures, ongoing glucocorticoid therapy, current smoking habits, and alcohol consumption [9]. Individuals with CKD or kidney failure face unique additional risk factors, as CKD leads to the onset of hypocalcemia, hyperparathyroidism, and vitamin D deficiency, which in turn results in reduced BMD [10]. Moreover, an extended duration of dialysis before transplantation raises the likelihood of BMD reductions [11]. The risk following transplantation involves the administration of glucocorticoids and ongoing hyperparathyroidism [12]. Moreover, the immunosuppressive treatment necessary following transplantation presents a particular risk for changes in bone turnover, mineralization, and volume [13]. Besides organ transplantation, various secondary factors leading to osteoporosis, such as rheumatoid arthritis, immobility, hyperparathyroidism, and others, increase the likelihood of experiencing osteoporosis [9, 14]. 

Although posttransplant bone disease poses distinct difficulties compared to pretransplant bone disease, these two may have a causal linkage. A recent investigation involving CKD patients revealed that reduced BMD at particular locations was significantly linked to overall mortality. The greatest risk for all-cause mortality was seen with reduced BMD assessed at the hip/femoral neck (69%), followed by the arm (26%), total body (25%), and spine (17%) [15]. According to Naylor et al.’s research, it has been revealed a wide range of fracture rates among KTRs, from 3.3 to 99.6 fractures per 1000 person-years [16]. As indicated in the recent meta-analysis released regarding fracture risk factors in KTRs in 2024, recipient age (hazard ratio [HR] = 1.50, 95% CI 1.17–1.91), female gender (HR = 1.45, 95% CI 1.36–1.53), diabetes before transplantation (HR = 1.76, 95% CI 1.58–1.97), prior fracture history before transplantation (HR = 2.28, 95% CI 1.86–2.78), length of dialysis (HR = 1.09, 95% CI 1.01–1.17), and utilizing a deceased donor (HR = 1.21, 95% CI 1.05–1.39) are associated with an increased fracture risk [17]. Hip fractures pose a significant issue, with research suggesting a 10–20% rise in mortality rates linked to these injuries. In the USA, around 25% of people with hip fractures need long-term home care, and the financial implications are substantial as well [18]. Screening-based prevention can be advantageous prior to transplantation by enhancing posttransplant outcomes related to bone health and hindering the onset of posttransplant bone complications. Information regarding the prevalence of osteoporosis among KTRs is limited. This study aims to estimate the prevalence of osteoporosis globally and regionally based on each skeletal site among KTRs.

## Methods

Using the available data, our research estimated the worldwide and continental prevalence of osteoporosis after KT. An ethical declaration is unnecessary for this research since it relies on a systematic review and meta-analysis of previously published literature. The methods for this research were performed in alignment with the 2020 PRISMA (Preferred Reporting Items for Systematic Reviews and Meta-Analyses) standards [19]. This review was registered with PROSPERO, CRD42024548222.

### Systematic search

We performed a systematic literature review to examine the prevalence of osteoporosis in adults after KT. We then conducted a meta-analysis to assess the overall prevalence of osteoporosis and to display the rates of the condition across various skeletal locations. We performed extensive electronic searches across multiple databases such as PubMed, Scopus, Web of Science, EMBASE, and Science Direct, along with the Google Scholar engine to find published research examining the prevalence of osteoporosis in patients who received KT from January 1, 2000, to January 1, 2024, in English.

### Search strategy

The search keywords were thoughtfully chosen from the Medical Subject Headings (MeSH) database, literature reviews, and other pertinent index terms. The search keywords comprised ‘Renal Transplantation,’ ‘Kidney Transplantation,’ ‘Renal Graft,’ ‘Kidney Graft,’ ‘Renal recipients,’ ‘Kidney recipients,’ ‘Renal transplant recipients,’ ‘kidney transplant recipients,’ ‘Osteoporosis,’ ‘Osteoporoses,’ ‘Bone Loss,’ ‘bone disease,’ ‘bone health,’ and ‘bone mineral density,’ with all potential word combinations customized to the unique patterns of each database. Furthermore, the search was improved by manually examining the reference lists of the identified articles to guarantee thoroughness. Further information about the study search is available in the supplementary file.

### Inclusion criteria

The criteria for inclusion in this study involved all research addressing osteoporosis in individuals aged 18 and above, identified by measuring BMD using standard equipment as per the International Society for Clinical Densitometry (ISCD, defined as a Z-score of ≤ − 2 for premenopausal women and men under 50, and a T-score of ≤ − 2.5 for postmenopausal women and men over 50) [20, 21], from January 1, 2000, to January 1, 2024. This comprised observational research like cross-sectional, case-control [with controls involving KT patients, healthy individuals, and deceased donors], cohort studies, studies assessing the baseline prevalence of osteoporosis based on specific bone site such as lumbar, femoral neck, total hip, forearm, and ultradistal radius. Nine articles were excluded from the screening process because of their unavailability [22–30].

### Exclusion criteria

The criteria for exclusion in this study involved duplicate studies, research unrelated to the topic and objectives of the study, studies with ambiguous methodology, interventional research without baseline osteoporosis reports, experimental and qualitative studies, case reports, and studies that are not in English. Furthermore, we excluded conference abstracts, protocols, books/book chapters, preprints, reviews (be it narrative or systematic), along with letters, news articles, opinions, and commentaries. Studies excluded during the full-text screening phase are summarized with brief explanations in the supplementary file, eTable 1.

### Selection process

Through utilizing reference management software, EndNote X7 (version 17), duplicate records removed. The eligibility of the studies was evaluated by reviewing their titles and abstracts. Subsequently, two researchers, M.G. and T.M., assessed the full texts separately. Any discrepancies were addressed with the involvement of the third author, M.M.

### Data collection process

Two researchers (M.G. and T.M.) independently gathered data using a structured and systematic data collection form. Any discrepancies among investigators were addressed, and a consensus was reached through discussions with the third author (M.M.). The subsequent information was collected from each investigation: author name, publication year, location, sample size, age, gender, diagnostic criteria for osteoporosis, HDI Tier, total number of osteoporosis across each skeletal site including lumbar, femoral neck, total hip, forearm, and ultradistal radius.

### Risk of bias assessment

In this study, two researchers (M.G. and M.M.) performed the risk of bias (ROB) assessment independently, with any differences addressed by the supervisor (T.M.) when needed. The quality of the articles was assessed using the JBI Critical Appraisal Checklist [31]. The JBI tool consists of nine items, each offering four possible answers: yes, no, unclear, or not applicable. A greater quantity of ‘yes’ responses signifies improved study quality. Further information regarding the methodology evaluation is available in the supplementary file, eTable 2.

### Definition

HDI Tier: In this research, we also investigated the prevalence of KTRs, based on the HDI scores of countries in 2024. The HDI developed by the United Nations Development Programme (UNDP) in 1990 and released annually, measures human development by focusing on ‘enlarging people’s choices.’ It captures the fundamental aspects of health, education, and income, which are the building blocks of these choices. A country's HDI value is calculated by considering a wide range of indicators such as life expectancy, literacy rate, access to electricity in rural areas, Gross Domestic Product (GDP) per capita, trade statistics, homicide rate, multidimensional poverty index, income inequality, and internet access. These indicators are combined to create a single value on a scale from 0 to 1.0, with 1.0 representing the highest level of human development [32, 33]. The HDI is categorized into four tiers based on the level of development: very high (0.8–1.0), high (0.7–0.79), medium (0.55–0.70), and low (below 0.55) (https://worldpopulationreview.com/country-rankings/hdi-by-country).

### Data analysis

The data analysis utilized Stata version 11 software to calculate the osteoporosis prevalence in KTRs, applying the binomial distribution formula. Heterogeneity was evaluated visually through forest plots. The Cochran’s Q test (using a significance level of 0.05) and *I*^2^ statistic (with *I*^2^ values of ≥ 50% indicating significant heterogeneity) were utilized to evaluate the significance of statistical heterogeneity [34]. The assessment of publication bias was conducted using a funnel plot and Egger's test. The Trim and Fill method was also utilized to assess publication bias. A random-effects model was used to determine the prevalence of osteoporosis in KTRs. The prevalence estimates with their 95% confidence intervals are shown in the forest plot diagram. Subgroup analyses conducted on the basis of continent, HDI category, different skeletal locations, and gender. Moreover, the meta-regression technique was utilized to identify the source of heterogeneity, incorporating continent and HDI tier into the meta-regression model. Furthermore, a sensitivity analysis was performed to evaluate the influence of each primary study on the overall estimate. It is important to note that the standards for identifying osteoporosis were mostly uniform among the primary studies, eliminating the necessity to examine this factor for heterogeneity.

For post hoc analysis, we considered subgroups based on the presence of adequate data regarding osteoporosis from eligible studies, which included studies that identified the donor type (living or deceased) of KTRs, simultaneous transplantation of KTRs with other solid organs, comorbid conditions such as pretransplant diabetes mellitus, pretransplant hypertension, pretransplant parathyroidectomy, menopausal status, and immunosuppressive medications including tacrolimus, cyclosporine, and mammalian target of rapamycin (mTOR) inhibitors. The overall prevalence of osteoporosis was determined using the inverse variance approach and a random-effects model (REM), with results presented alongside a 95% confidence interval. Additionally, research that detailed the mean and standard deviation for quantitative variables was included such as age, BMI, time posttransplantation, and hemodialysis duration—prior to transplantation between KTR adults with osteoporosis (case group) compared to KTRs with normal BMD (control group). According to the T-score classifications, the BMD was classified as osteopenia (T-score between − 1.0 and − 2.5), osteoporosis (T-score of − 2.5 or lower), and normal BMD (T-score of − 1.0 or higher) [20, 21]. For the studies included that reported median and interquartile range or median and range, we applied the techniques described by Luo et al. and Wan et al. [35, 36] to obtain mean and standard deviation. Based on the quantitative data, the standardized mean difference (SMD) was calculated. We analyzed the SMD, participant number, means, and standard deviations of the quantitative data for both case and control groups individually. Utilizing Methane's equation, a random-effects model, and Cohen's d coefficient, the SMD was calculated for each variable, accompanied by a 95% confidence interval. The criteria for evaluating the importance of the SMD of the results between two groups (case and control) are that zero must not be present within the upper and lower confidence intervals of the SMD [37].

## Result

### Study selection

**Fig. 1.**
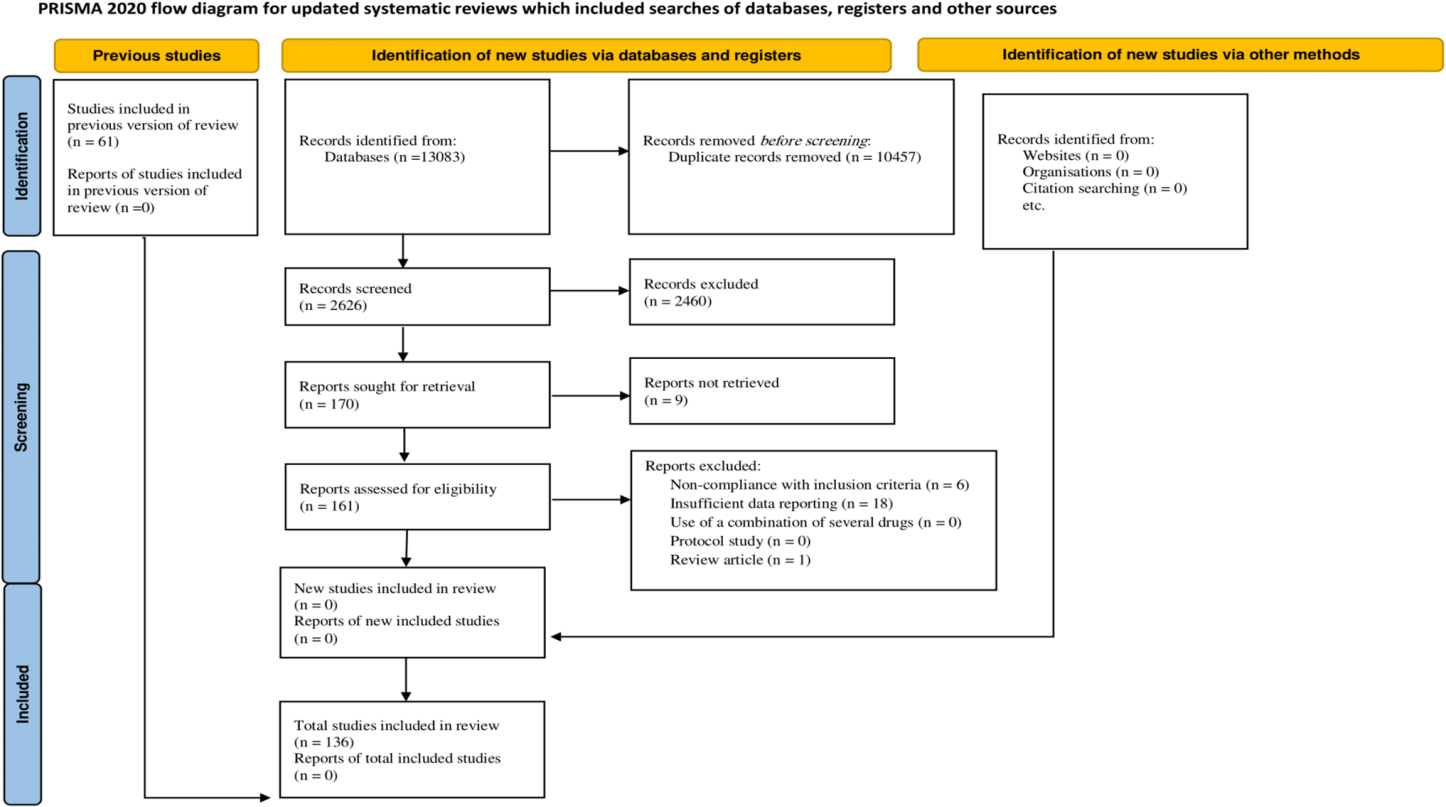
Flow diagram of included/excluded studies

Upon conducting comprehensive searches across various databases using relevant keywords, we initially identified 13,083 primary studies. Duplicate articles were initially flagged and removed using End Note software (*n* = 10,457). Subsequently, the titles and abstracts of the records were screened to eliminate irrelevant content (*n* = 2460). The next phase included exclusion of reports that were not retrieved (*n* = 9). Additionally, we excluded 25 studies for various reasons, including six for noncompliance with inclusion criteria, 18 for insufficient data reporting, and one review article. List of excluded studies with brief reasons is provided in supplementary file, eTable 1. Through a rigorous screening process and the application of specific inclusion and exclusion criteria, a total of 136 primary studies were ultimately included. Information essential to the study was then extracted from these articles. The selection process of the included studies is visually depicted in the PRISMA diagram (Fig. 1).

### Study characteristics

The included studies comprise 136 studies carried out in 38 countries and across five continents. The allocation of studies by continent is as such: 61 in Europe, 52 in Asia, 16 in the Americas, 4 in Africa, and 3 in Australia. These studies encompassed 45,254 KTRs, comprising 26,933 males and 17,308 females. Moreover, 17 studies offered information regarding the gender of the osteoporotic cases. According to the HDI Tier, 105 studies were categorized in the ‘Very High’ group, 26 studies were assigned to the ‘High’ group, and five studies were included in the ‘Medium’ group. None of the studies mentioned were carried out in nations with a low HDI. The characteristics of the studies that were included are available in the supplementary file, eTables 3 and 4.

### Prevalence of osteoporosis in the lumbar region

The prevalence of lumbar osteoporosis among KTRs is supported by 67 studies. Of these, 35 studies are from Europe, 22 from Asia, five from America, three from Africa, and two from Australia. Among these, 51 studies are from countries with very high HDI, 15 from countries with high HDI, and one from a country with medium HDI. The prevalence of lumbar osteoporosis among KTRs ranges from 4.1% in Sun's study to 82.4% in Brunova et al.'s study. Heterogeneity indices (I-squared: 89.94%, Q: 656.29, *P* < 0.001) indicate a high heterogeneity in the results of primary studies. Through the synthesis of data from 67 studies, it is estimated that the prevalence of osteoporosis in the lumbar region is 20% (95% CI 18–23) (Table 2 and efigure 1). Subgroup analysis by continent showed that the prevalence of lumbar osteoporosis was highest in Europe at 23% (95% CI 20–27), followed by Asia at 19% (95% CI 14–24), America at 14% (95% CI 8–20), Africa at 12% (95% CI 7–17), and Australia at 9% (95% CI 5–14) (Table 2 and efigure 2). In countries with very high HDI, the prevalence is higher at 22% (95% CI 19–25), whereas in countries with high HDI, it is lower at 11% (95% CI 8–14). The prevalence reported in a country with medium HDI was 33% (95% CI 24–45) (Table 2 and efigure 3). The publication bias is indicated by the funnel plot (efigure 4) and Begger's test (*β* = 4.07, *P* < 0.001). To address this bias, a trim and fill analysis was conducted (efigure 5). Based on this analysis, a new study has not been evaluated and the prevalence of osteoporosis in the lumbar region remains unchanged. The meta-regression test, which was performed to investigate the factors related to the heterogeneity, showed that the continent (*β* = 0.03, *P* = 0.052) and the HDI level (*β* = − 0.07, *P* = 0.048) were not related to the heterogeneity. It is important to mention that, according to the findings of the sensitivity analysis, the influence of each primary study on the overall estimate was not significant. Additionally, the findings of the sensitivity analysis regarding the impact of KTR studies involving deceased donors on the overall estimate were not significant. The meta-analysis results and subgroup analysis for lumbar region are detailed in Table 1.

**Table 1.** Summary of meta-analysis and subgroups analysis results according to the reported osteoporosis for each bone site

Bone site/continents or HDI tier	No. of studies (*n*)	Cases (*n*)^a^	Prevalence rate		Heterogeneity		
			ES (95% CI)	Model	Chi-square	*P* value	*I* square (%)
*Lumbar osteoporosis*							
Europe	35	1015	23% (95% CI 20–27)	Random	397.33	< 0.001	91.44%
Asia	22	374	19% (95% CI 14–24)	Random	187.59	< 0.001	88.81%
America	5	108	14% (95% CI 8–20)	Random	22.42	< 0.001	82.16%
Africa	3	20	12% (95% CI 7–17)	Random	–	–	–
Australia	2	17	9% (95% CI 5–14)	Random	–	–	–
Very high HDI	51	1374	22% (95% CI 19–25)	Random	577.64	< 0.001	91.34%
High HDI	15	135	11% (95% CI 8–14)	Random	31.87	< 0.001	56.08%
Medium HDI	1	25	33% (95% CI 24–45)	Random	–	–	–
Overall	67	1534	20% (95% CI 18–23)	Random	656.29	< 0.001	89.94%
*Femoral neck osteoporosis*							
Europe	28	902	23% (95% CI 19–27)	Random	275.26	< 0.001	90.19%
Asia	18	437	24% (95% CI 17–32)	Random	375.87	< 0.001	95.48%
America	4	81	16% (95% CI 6–25)	Random	34.22	< 0.001	91.23%
Africa	2	14	14% (95% CI 7–21)	Random	–	–	–
Australia	1	26	18% (95% CI 12–25)	Random	–	–	–
Very high HDI	41	1232	22% (95% CI 19–26)	Random	522.48	< 0.001	92.34%
High HDI	11	183	20% (95% CI 11–29)	Random	162.36	< 0.001	93.84%
Medium HDI	1	45	60% (95% CI 49–70)	Random	–	–	–
Overall	53	1460	23% (95% CI 19–26)	Random	765.85	*P* < 0.001	93.21%
*Total hip osteoporosis*							
Europe	8	235	23% (95% CI 15–31)	Random	107.67	< 0.001	93.50%
Asia	7	53	9% (95% CI 6–13)	Random	9.76	< 0.001	38.55%
America	3	68	12% (95% CI 5–20)	Random	–	–	–
Africa	1	3	5% (95% CI 2–14)	Random	–	–	–
Australia	1	3	10% (95% CI 3–26)	Random	–	–	–
Very high HDI	17	354	17% (95% CI 13–21)	Random	148.09	< 0.001	89.20%
High HDI	3	8	5% (95% CI 1–9)	Random	–	–	–
Medium HDI	–	–	–	–	–	–	–
Overall	20	362	15% (95% CI 11–19)	Random	159.59	< 0.001	88.09%
Ultradistal radius osteoporosis	6	360	34% (95% CI 28–40)	Random	18.34	< 0.001	72.74%
Forearm osteoporosis	5	79	29% (95% CI 10–49)	Random	97.07	< 0.001	95.88%

*HDI* human development index

^a^This column represents the number of patients used to calculate the prevalence rate

### Prevalence of osteoporosis in the femoral neck region

The prevalence of osteoporosis in the femoral neck has been reported in 53 studies. Of these, 28 studies are from Europe, 18 from Asia, four from America, two from Africa, and one from Australia. Out of these, 41 studies are related to countries with very high HDI, 11 studies are related to countries with high HDI, and one study is related to medium HDI. The prevalence of osteoporosis in the femoral neck varied widely, ranging from 3.2% in both Wong’s and Sun’s study to 78.1% in Alfieri et al.'s study. Heterogeneity indices indicate a high level of heterogeneity between the results of primary studies (I-squared: 93.21%, Q: 765.85, *P* < 0.001). Through the combination of findings of the 53 studies, the estimated prevalence of osteoporosis in the femoral neck is 23% (95% CI 19–26) (Table 2 and efigure 6). Based on subgroup analysis by continent, the prevalence of femoral neck osteoporosis was highest in Asia at 24% (95% CI 17–32), followed by Europe at 23% (95% CI 19–27), America at 16% (95% CI 6–25), Africa at 14% (95% CI 7–21), and Australia at 18% (95% CI 12–25) (Table 2 and efigure 7). Specifically, in countries with very high HDI, the prevalence is 22% (95% CI 19–26), while in countries with high HDI, it is estimated at 20% (95% CI 11–29). The prevalence reported in a country with medium HDI was estimated to be 60% (95% CI 49–70) (Table 2 and efigure 8). Furthermore, the presence of publication bias is indicated by the funnel plot (efigure 9) and Begger's test (*β* = 4.67, *P* < 0.001). To address this bias, a trim and fill analysis was performed (efigure 10). According to the results of this analysis, a new study has not been incorporated and the prevalence of osteoporosis remains unchanged. The meta-regression test, aimed at exploring factors contributing to heterogeneity, revealed that the continent (*β* = 0.01, *P* = 0.427) and the HDI level (*β* = 0.03, *P* = 0.482) were not associated with heterogeneity. It is essential to highlight that, according to the results of the sensitivity analysis, the impact of each primary study on the overall estimate was not significant. Additionally, the results of the sensitivity analysis regarding the impact of KTR studies with deceased donors on the overall estimate were not significant. The meta-analysis results and subgroup analysis for femoral neck region are detailed in Table 1.

**Table 2.** Post hoc subgroup analysis

Characteristics	No. of studies/total studies	Cases (n)	Prevalence rate		Heterogeneity		
			ES (95%CI)	Model	Chi-square	*P* value	I-square (%)
*Osteoporosis region, donor type*^a^							
General, living^b^	7/75	166	11% (95% CI 5–17)	Random	66.2	< 0.001	90.94%
Lumbar, deceased^c^	11/67	371	26% (95% CI 18–34)	Random	140.76	< 0.001	92.9%
Femoral neck, deceased^d^	10/53	295	23% (95% CI: 15–32)	Random	173.5	< 0.001	94.81%
Total hip, deceased^e^	3/20	63	10% (95% CI 3–17)	Random	7.36	0.03	72.81%
Forearm, living^f^	3/5	26	14% (95% CI 0–28)	Random	21.09	< 0.001	90.52%
Simultaneous transplantation^g^	3/75	61	38% (95% CI 3–74)	Random	70.3	< 0.001	97.16%
*Comorbidities*							
Diabetes mellitus^h^—pretransplant	11/136	104	29% (95% CI 19–39)	Random	60.72	< 0.001	83.53%
Hypertension^I—^pretransplant	6/136	102	26% (95% CI 12–40)	Random	52.55	< 0.001	90.49%
Parathyroidectomy^j^—pretransplant	4/136	19	12% (95% CI 6–19)	Random	3.95	0.27	24.08%
Menopausal^k^	10/136	99	32% (95% CI 24–41)	Random	26.22	< 0.001	65.68%
*Immunosuppressive treatments*							
Tacrolimus^l^	9/136	126	14% (95% CI 9–19)	Random	49.94	< 0.001	83.98%
Cyclosporine^m^	8/136	123	17% (95% CI 9–25)	Random	40.6	< 0.001	82.76%
mTOR inhibitors^n^	5/136	88	22% (95% CI 10–34)	Random	39.72	< 0.001	89.93%

*KTR* kidney transplant recipients, *mTOR* mTOR: mammalian target of rapamycin

^a^This subgroup provides general and each bone site osteoporosis based on the donor type (living or deceased) of KTRs patients

^b^This subgroup provides prevalence rate for general osteoporosis among KTRs with living donor. Summary of the findings is provided in eTable 5 and eFig 27

^c^This subgroup provides prevalence rate for lumbar osteoporosis among KTRs with deceased donor. Summary of the findings is provided in eTable 5 and eFig 28

^d^This subgroup provides prevalence rate for femoral neck osteoporosis among KTRs with deceased donor. Summary of the findings is provided in eTable 5 and eFig 29

^e^This subgroup provides prevalence rate for total hip osteoporosis among KTRs with deceased donor. Summary of the findings is provided in eTable 5 and eFig 30

^f^This subgroup provides prevalence rate for forearm osteoporosis among KTRs with living donor. Summary of the findings is provided in eTable 5 and eFig 31

^g^This subgroup includes studies with simultaneous kidney transplantation with other organs, which two studies including pancreas–kidney transplantation [38, 39] and one study including liver‑kidney transplantation [40]. Summary of the findings is provided in eTable 5 and eFig 32

^h^Prevalence of osteoporosis among KTRs with pretransplant diabetes mellitus. Summary of the findings is provided in eTable 5 and eFig 33

^i^Prevalence of osteoporosis among KTRs with pretransplant hypertension. Summary of the findings is provided in eTable 5 and eFig 34

^j^Prevalence of osteoporosis among KTRs with pretransplant parathyroidectomy. Summary of the findings is provided in eTable 5 and eFig 35

^k^Prevalence of osteoporosis among postmenopausal female KTRs. Summary of the findings is provided in eTable 5 and eFig 36

^l^Prevalence of osteoporosis among KTR patients receiving tacrolimus. Summary of the findings is provided in eTable 5 and eFig 37.

^m^Prevalence of osteoporosis among KTR patients receiving cyclosporine. Summary of the findings is provided in eTable 5 and eFig 38.

^n^Prevalence of osteoporosis among KTR patients receiving mTOR inhibitors. Summary of the findings is provided in eTable 5 and eFig 39.

### Prevalence of osteoporosis in the total hip region

In 20 studies, the prevalence of osteoporosis in the total hip region has been examined. Of these, eight studies are from Europe, seven from Asia, three from America, one from Africa, and one from Australia. Of these studies, 17 were conducted in countries with very high HDI, while three were from countries with high HDI. The prevalence of osteoporosis in the total hip varied widely, ranging from 3.2% in Wong's study to 56% in Rubello et al.'s study. Heterogeneity indices (I-squared: 88.09%, Q: 159.59, *P* < 0.001) indicated high heterogeneity among the primary studies. Combining the findings of these 20 studies, the estimated prevalence of osteoporosis at the total hip region is 15% (95% CI 11–19) (Table 2 and efigure 11). Based on continent-specific subgroup analysis, the prevalence of lumbar osteoporosis was highest in Europe at 23% (95% CI 15–31), followed by Asia at 9% (95% CI 6–13), America at 12% (95% CI 5–20), Africa at 5% (95% CI 2–14), and Australia at 10% (95% CI 3–26) (Table 2 and efigure 12). The prevalence of osteoporosis in the total hip is 17% (95% CI 13–21) in countries with very high HDI, and 5% (95% CI 1–9) in countries with high HDI (Table 2 and efigure 13). Publication bias is indicated by the funnel plot (efigure 14) and Begger's test (*β* = 3.44, *P* < 0.009). To address this bias, a trim and fill analysis was conducted (efigure 15). Based on the findings of the study, a new study has not been evaluated and the prevalence of osteoporosis in the total hip region remains unchanged. The meta-regression test revealed that the continent (*β* = 0.05, *P* = 0.051) and HDI level (*β* = − 0.11, *P* = 0.181) were not associated with heterogeneity. Results from the sensitivity analysis indicate that the influence of each primary study on the overall estimate was not significant. Additionally, the results of the sensitivity analysis regarding the influence of KTR studies with deceased donors on the overall estimate were not significant. The meta-analysis results and subgroup analysis for total hip region are detailed in Table 1.

### Prevalence of osteoporosis in the forearm region

In five studies, the prevalence of osteoporosis in the forearm region has been examined. The heterogeneity indices indicate a high level of heterogeneity between the results of the primary studies (I-squared: 95.88%, Q: 97.07, *P* < 0.001). Combining the results of these studies, the estimated prevalence of osteoporosis in the forearm is 29% (95% CI 10–49) (Table 2 and eFigure 16). According to the sensitivity analysis, the effect of each primary study on the overall estimate was not significant. Furthermore, the results of the sensitivity analysis regarding the impact of KTR studies with living donors on the overall estimate were not significant. The meta-analysis result for forearm region is presented in Table 1.

### Prevalence of osteoporosis in the ultradistal radius region

In six studies, the prevalence of osteoporosis in the ultradistal radius has been documented. The heterogeneity indices (I-squared: 72.74%, Q: 18.34, *P* < 0.001) indicate high heterogeneity among the primary study results. A combined analysis of these studies estimates that the prevalence of osteoporosis in the ultradistal region is 34% (95% CI 28–40) (Table 2 and eFigure 17). Sensitivity analysis showed that the each primary studies did not significantly affect the overall estimate. The result of the meta-analysis for the ultradistal radius region is presented in Table 1.

### Prevalence of general osteoporosis

In 75 studies, the prevalence of general osteoporosis [general osteoporosis; encompassing those studies that did not specify osteoporosis cases based on each bone site and or did not specify osteoporosis definition] was reported. Among these, 32 studies developed from Asia, 30 from Europe, 11 from America, one from Africa, and one from Australia. The prevalence of general osteoporosis among KTRs ranged from 1.3% in Roberts' study to 75% in Rocha et al.'s study. Heterogeneity indices (I-squared: 97.01%, Q: 2477.4, *P* < 0.001) indicated high heterogeneity among the results of primary studies. The combined results of 75 studies estimate that the prevalence of general osteoporosis among KTRs is 23% (95% CI 21–25) (eFigure 18). Furthermore, the publication bias is indicated by the funnel plot diagram (eFigure 19) and Begger's test (*β* = 4.72, *P* < 0.001). To address this bias, a trim and fill analysis was conducted (eFigure 20). Based on this test, a total of 21 new studies have been included, the general prevalence of osteoporosis among the individuals is estimated to be 16% (95% CI 14.2–17.9).

### Prevalence of osteoporosis in male patients

In 16 studies, the prevalence of osteoporosis among male KTRs has been thoroughly examined. The prevalence of osteoporosis in men has shown significant variation, ranging from 4.9% in Lin's study to 85% in Rocha’s study. Heterogeneity indices (I-squared: 93.15%, Q: 218.82, *P* < 0.001) indicate high heterogeneity among the results of these primary studies. Upon combining the findings of these 16 studies, the estimated prevalence of osteoporosis among male KTRs is 21% (95% CI 15–27) (eFigure 21). Both the funnel plot (eFigure 22) and Begger's test (*β* = 4.86, *P* < 0.001) revealed publication bias. To address this bias, a trim and fill analysis was conducted (eFigure 23). Based on the findings of this analysis, two new studies have been considered that the prevalence of osteoporosis among male KTRs changed to 15.5% (95% CI 9–22.1). The meta-regression test, conducted to explore factors contributing to heterogeneity, revealed that the continent (*β* = 0.01, *P* = 0.927) and the HDI level (*β* = 0.15, *P* = 0.327) were not associated with heterogeneity. The results of the sensitivity analysis indicated that the influence of each primary study on the overall estimate was not significant.

### Prevalence of osteoporosis in female patients

In 16 studies, the prevalence of osteoporosis among female KTRs has been investigated. The prevalence of osteoporosis in women has shown significant variation, ranging from 6.3% in Gupta’s study to 68% in Rocha’s study. The heterogeneity indices (I-squared: 91.40%, Q: 174.41, *P* < 0.001) indicate high heterogeneity in the results of the primary studies. Combining the findings of these 16 studies, it is estimated that the prevalence of osteoporosis among female KTRs is 28% (95% CI 20–35) (Table 1 and eFigure 24). Both the funnel plot (eFigure 25) and Begger's test (*β* = 4.45, *P* < 0.001) suggest a publication bias in estimating the prevalence of osteoporosis among female KTRs. To address this bias, a trim and fill analysis was conducted (eFigure 26). Based on this analysis, the prevalence of osteoporosis in female KTRs remains unchanged. The meta-regression test revealed that the continent (*β* = 0.03, *P* = 0.586) and HDI level (*β* = 0.22, *P* = 0.092) were not associated with heterogeneity. It is essential to mention that the sensitivity analysis showed that the impact of each primary study on the overall estimate was not significant.

### Post hoc subgroup analysis

#### Prevalence of osteoporosis among KTRs stratified by donor type, simultaneous transplantation, comorbidities, and immunosuppressive treatments

From the available data across the 136 studies included, several articles provided sufficient information to compute the prevalence of osteoporosis according to donor type (living or deceased), simultaneous transplantation, comorbidities, and immunosuppressive therapies.

Based on the donor type, the prevalence of osteoporosis in the lumbar spine, femoral neck, and total hip among KTRs with deceased donors were 26% (95% CI 18–34; KTRs (*n*): 1603, Male/Female: 934:669), 23% (95% CI 15–32; KTRs (*n*): 1523, Male/Female: 891:632), and 10% (95% CI 3–17; KTRs (*n*): 573, Male/Female: 351:222), respectively. The prevalence of forearm osteoporosis in KTRs with living donors was 14% (95% CI 0–28; KTRs (*n*): 160, Male/Female: 160:0). The prevalence of general osteoporosis in KTRs with living donors was 11% (95% CI 5–17).

The prevalence of osteoporosis related to concurrent transplantation of the kidney along with other solid organs such as the pancreas and liver was 38% (95% CI 3–74).

Based on comorbid conditions, the highest prevalence of osteoporosis was found in postmenopausal female KTRs (32%, 95% CI 24–41), followed by KTRs who had pretransplant diabetes (29%, 95% CI 19–39), KTRs with pretransplant hypertension (26%, 95% CI 12–40), and KTRs who underwent pretransplant parathyroidectomy (12%, 95% CI 6–19).

According to immunosuppressive therapies, the prevalence of osteoporosis in KTRs treated with tacrolimus, cyclosporine, and mTOR inhibitors (including sirolimus and everolimus) was 14% (95% CI 9–19), 17% (95% CI 9–25), and 22% (95% CI 10–34), respectively. The findings of the included studies and results of the post hoc subgroup analysis are outlined in eTable 5 and Table 2, respectively.

#### Standardized mean difference of the clinical findings among KTR adults with osteoporosis compared to KTRs with normal BMD

According to the data availability, several articles supplied sufficient data for a comparative analysis of the SMD in clinical findings between KTRs with osteoporosis and those possessing normal BMD. A random-effects meta-analysis was performed on the clinical findings' values, which included age, BMI (kg/m2), time since transplantation (months), and pretransplant duration of hemodialysis (months). In KTRs with osteoporosis, age [SMD = 0.28, 95% CI 0–0.57, *P* < 0.001] and the duration of hemodialysis [SMD = 0.18, 95% CI 0–0.36, *P* = 0.058] were significantly higher compared to individuals with normal BMD. However, in KTRs with osteoporosis, BMI [SMD = − 0.56, 95% CI − 0.71 to − 0.41, *P* = 0.015] was significantly lower than those with normal BMD. The findings of the included studies and results of the SMD for the clinical data are outlined in eTable 5 and Table 3, respectively.

**Table 3.** Summary of meta-analysis of the SMD for the clinical findings among KTR adults with osteoporosis compared to KTRs with normal BMD

Characteristics	No. of studies/total studies	Cases versus controls	Mean values of cases versus controls, 95% CI	Model	SMD, 95% CI	Heterogeneity		
						Chi-square	*P* value	I-square (%)
Age, years^a^	20/136	647 vs. 1295	47.5, 42.5 to 52.5 vs. 44.7, 41.6 to 47.8	Random	0.28, 0 to 0.57	111.01	< 0.001	82.9%
Body mass index (kg/m^2^)^b^	20/136	661 vs. 1475	22.96, 22.31 to 23.62 vs. 25.33, 24.69 to 25.97	Random	− 0.56, − 0.71 to − 0.41	34.72	0.015	45.3%
Time since transplantation (months) ^c^	12/136	327 vs. 708	56.31, 44.02 to 68.59 vs. 55.42, 45.05 to 65.79	Random	0.01, − 0.14 to 0.15	10.65	0.473	0%
Duration of hemodialysis—pretransplant (months) ^d^	10/136	454 vs. 858	35.27, 26.92 to 43.63 vs. 30.76, 22.78 to 38.75	Random	0.18, 0 to 0.36	16.46	0.058	45.3%

^a^Summary of the SMD is provided in eTable 5 and eFig 40

^b^Summary of the SMD is provided in eTable 5 and eFig 41

^c^Summary of the SMD is provided in eTable 5 and eFig 42

^d^Summary of the SMD is provided in eTable 5 and eFig 43

### Risk of bias assessment

Out of 136 studies, 45 were found to have a medium risk of bias, while the number of studies classified as having low and high risk of bias was 8 and 83, respectively. Detailed assessments of the ROB can be found in the Supplementary file, eTable 2.

## Discussion

This meta-analysis represents a pioneering effort to evaluate the prevalence of osteoporosis among KTRs. The heterogeneity of the studies in the meta-analysis is significant and may have affected the results. Potential sources of heterogeneity among studies were examined through prespecified subgroup analysis based on continent and HDI tier. The absence of a significant link between HDI tier and continent regarding osteoporosis prevalence could stem from the insufficient number of studies performed in nations with high, medium, and low HDI, along with low number of studies conducted in the continents of America, Africa, and Australia, potentially causing this lack of significance. The high heterogeneity may stem from variations in demographic factors and clinical characteristics of the samples analyzed; the limited number of studies incorporated in subgroup and meta-regression analysis could result in an inability to identify the source of heterogeneity. A sensitivity analysis utilizing a random-effects model carried out by removing one individual study at a time indicated no alterations in the pooled analyses, suggesting the statistical reliability of the results. The importance of considering these factors for osteoporosis is that the osteoporosis prevalence can be influenced by the population density, age, economic circumstances, variations in the quality of medical service delivery, availability of osteoporosis screening options, and management of potential risk factors. Considering the significant clinical, economic, and social impacts of osteoporosis and the aging demographic, it is crucial for healthcare providers and policymakers worldwide to comprehend the prevalence of osteoporosis and the association of these factors to osteoporosis prevalence. This information could also enhance the estimation of the number of DXA scanners needed for identifying osteoporosis cases and for the ongoing assessment of disease advancement and treatment efficacy in any specific country or region.

The dominance and elevated prevalence rates of osteoporosis in Europe and Asia were primarily shaped by a greater number of studies carried out in these regions compared to other continents (e.g., Africa, America, and Australia). The findings illustrate that osteoporosis is a prevalent condition in certain developed nations across America, Europe, and Asia. Bone site-specific osteoporosis indicated that the prevalence of lumbar, femoral neck, and total hip osteoporosis was greatest in countries with very high HDI. According to continent level for each bone site, the prevalence of lumbar and total hip osteoporosis was greatest in Europe, whereas femoral neck osteoporosis was most prevalent in Asia. The lumbar spine and femoral neck are the most common sites for BMD evaluation [41]. Our findings indicated that the femoral neck was more prone to osteoporosis compared to the lumbar spine. The prevalence of osteoporosis was elevated when diagnosed at these sites in KTRs; we recommend that the spine and femoral neck should be assessed concurrently for osteoporosis diagnosis in KTRs, following the recommendation from the International Society for Clinical Densitometry (ISCD) [42]. Considering the mean age data of osteoporotic patients (47.5 years), these findings indicate that the spine and femoral neck of KTR patients ought to be eligible for DXA scans earlier in life compared to non-CKD individuals, as suggested by ISCD.

The prevalence of osteoporosis is increasing worldwide, especially in countries with a high sociodemographic index (SDI) [43]. It has recently been demonstrated that nations or areas experiencing a greater incidence of fractures linked to low BMD exist in both economically developed and less affluent regions. Additionally, nations and areas with middle and low-middle SDI levels demonstrated a trend of swift increase [44]. A study of the global and regional prevalence of osteoporosis according to the WHO criteria in 2022, the prevalence rate of osteoporosis among developed and developing countries was reported to be 14.5% (95% CI 11.5–17.7) and 22.1% (95% CI 20.1–24.1), respectively [45]. In industrialized countries with very high HDI (France, Germany, Italy, Spain, UK, USA, Canada, Japan, and Australia), the number of people with osteoporosis are estimated at 49 million. The highest prevalence of osteoporosis in the spine or hip was reported 26.3% in Japan, followed by 21% in the USA, 14.3% in Germany, 9.9% in France, 9.7% in Italy, 7.8% in the UK, 6.3% in Spain, 2.6% in Canada, and 2% in Australia [46]. While our study had a limited number of studies with medium HDI, the prevalence rates of lumbar, femoral neck, and total hip osteoporosis were significant in countries with high and very high HDI. Nations with very high HDI tier also exhibited the greatest prevalence rates in particular skeletal sites, with 22% in the lumbar region, 22% in the femoral neck region, and 17% in the total hip region. The causes for this outcome could be linked to elements like the significant frequency of healthcare visits in regions with high HDI and the extended average lifespan of the people. The heightened burden of disease seen in developed nations can be linked to their considerably larger populations.

The highest prevalence of osteoporosis after KT was found in bone sites abundant in cortical content (e.g., ultradistal radius). In line to our study, the highest prevalence of low BMD (T‑score ≤ − 2.5) in the whole CKD patients was at the distal radius (31%; 95% CI 20–43), followed by femoral neck (19%; 95% CI 16–23), and hip (18%; 95% CI 11–27) [4]. The elevated prevalence of osteoporosis at these locations greatly affects the prognosis of these patients, heightening their vulnerability to fractures and the likelihood of death. In patients with CKD, decreased BMD at the hip/femoral neck is linked to the greatest risk of overall mortality (pooled RR = 1.69; 95% CI 1.20–2.40). Moreover, the mortality risk for each standard deviation reduction in BMD is notably greater at the hip/femoral neck (pooled RR = 1.43, 95% CI 1.15–1.77) than at the arm (pooled RR = 1.03, 95% CI 1.00–1.06) and spine (pooled RR = 1.17, 95% CI 0.98–1.39) [15]. In comparison to BMD readings at other anatomical locations, BMD assessed in the hip area might serve as the most reliable indicator of mortality risk in CKD patients, especially those with ESRD or on dialysis. BMD in the hip area might more accurately represent bone disease and metabolic changes in CKD patients and appears to be especially significant for tracking CKD outcomes [47, 48]. Given that insufficient research thoroughly evaluates the connection between BMD at different bone locations and the risk of all-cause mortality in KTRs, the osteoporosis prevalence rates of the current research for each bone site are vital for understanding and guiding current clinical practices in managing KTRs to reduce possible morbidity and mortality.

Our research indicated that the estimated prevalence of osteoporosis in female patients was 7% greater than in male patients, based on the sex-specific osteoporosis rates reported in the studies considered. Additionally, the overall prevalence of osteoporosis in postmenopausal women was greater than in men (32%, 95% CI 24–41 compared to 21%, 95% CI 15–27). Additionally, based on the earlier meta-analysis on fracture risk factors in KTRs released in 2024, being female (hazard ratio [HR] = 1.45, 95% CI 1.36–1.53) was linked to an increased risk of fractures [17]. This matter suggests that females face a higher risk of bone loss compared to males. The prevalence of low BMD (T-score ≤ − 2.5) categorized by gender across the entire range of CKD showed a prevalence of 32% (95% CI 24.9–39.2; 11,177 participants) in females and 23% in males (95% CI 16.0–31.6; 10,494 participants) [4]. In line to our study, the global prevalence of osteoporosis in 2022 revealed gender-specific rates, indicating that the prevalence in women was 24.8% (95% CI, 22.5–27.3) and in men was 10.6% (95% CI 9.1–12.1), respectively [45]. Studies consistently indicate that, in the general population, women experience a greater prevalence of bone mineral disease than men. This is attributed to variations in BMD between men and women at maturity, along with the pace of cortical bone loss in both sexes.

Besides estrogen deficiency and parity, which are closely linked to osteoporosis in women and contribute to its higher prevalence in females compared to males, the distribution of factors like obesity, diabetes, and metabolic syndrome varies between sexes, potentially influencing BMD and leading to differences in osteoporosis prevalence between males and females [49, 50].

Along with estrogen, testosterone may reduce bone resorption, providing another explanation for the decreased osteoporosis risk in males compared to females. The decline in plasma estrogen levels in postmenopausal women may also clarify why these women are more susceptible to osteoporosis compared to their premenopausal phase. Before the menopausal transition, they experience an average annual bone loss of 2%. Nevertheless, in the transmenopause phase, starting roughly 2 years prior to a woman's last menstrual period and continuing for several years thereafter, women experience an average yearly bone loss rate of 10–12% in both the hip and spine. Subsequently, the pace of bone loss significantly decreases to an average of 0.5% annually [51, 52]. Following the age of 50, there is a notable decline in muscle mass accompanied by similar gender-neutral alterations such as heightened satellite cell senescence and inflammation, reduced protein synthesis and myocyte regeneration, along with various other gender-specific changes due to the reduction of sex hormones [53]. Reduced sex hormones in both sexes, commonly noted in the natural progression of CKD, may also influence the higher prevalence of low BMD in comparatively young KTRs [54]. Brandenburg et al. confirmed the association of low estradiol and elevated luteotropic hormone (LH) levels with yearly BMD decline in postmenopausal women who have undergone KT, resulting in a reduction of lumbar T-scores in the later stages posttransplant [55]. Circulating sex hormones significantly influence lumbar BMD, as shown by the negative relationship between vertebral column BMD and parathyroid hormone (PTH) in premenopausal women who have undergone renal transplantation [56]. Our research cannot obviously confirm that gender is a major factor for osteoporosis due to the sparse age data on KTRs with osteoporosis available in existing literature.

In KTRs, the presence of immunosuppressive drugs, low BMI levels, and additional conditions like diabetes may lead to an earlier and more pronounced decrease in expected BMD [57, 58]. Immunosuppressive drugs, such as calcineurin (CN) inhibitors and mTOR inhibitors, can affect bone metabolism [13]. Among CN inhibitors, the prevalence of osteoporosis noted in KTR patients treated with tacrolimus and cyclosporine was 14% (95% CI 9–19) and 17% (95% CI 9–25), respectively. Research on animals has shown that tacrolimus enhances bone volume and decreases the number of osteoclasts [59]. Nevertheless, comparative research in rodents has shown that, although cyclosporine can promote both bone formation and resorption, causing high-turnover bone loss, tacrolimus mainly increases bone resorption, leading to bone loss [60]. Evaluating the direct effect of these agents on bone health is more complex since the severity of bone disorder after transplantation is associated with the debilitating characteristics of the disease prior to transplantation [61]. Given these results, BMD monitoring is recommended in patients treated with CN inhibitors, as these drugs have shown dose-dependent impacts on bones in preclinical studies [60]. Conversely, sirolimus and everolimus are macrolide immunosuppressants that block mTOR, impacting T- and B-lymphocyte reactions to cytokines, in contrast to previous immunosuppressants like cyclosporine and tacrolimus [62]. The connection between mTOR inhibitors use and reduced BMD is a debated topic. Clinical research, especially in oncology, indicates that mTOR inhibition by everolimus reduces bone turnover regardless of previous therapies or bone metastases [63]. In KTRs, immunosuppression using sirolimus is associated with lower bone turnover markers and reduced osteoclast formation, providing an edge over CN inhibitors [64]. In vitro research indicates that sirolimus exhibits anti-proliferative properties in osteoblasts and osteoclasts, and it also aids in the final differentiation of osteoclasts [65–67]. The overall outcome of these effects is higher bone turnover and a reduction in bone mass. Additionally, mTOR inhibitors may delay the healing time following a bone fracture [67]. In alignment with our research, Gregorini et al. discovered that the use of sirolimus was a risk factor for the osteoporosis in KTRs [68]. Therefore, additional studies are required to comprehensively grasp the effects of mTOR inhibitors on the skeleton.

Our meta-analysis indicated that the BMI of osteoporotic patients (23 kg/m^2^) was significantly lower than those with normal BMD (25.3 kg/m^2^), demonstrating a moderate to large effect. Epidemiological data in the general population indicates that elevated body weight or high BMI is associated with increased bone density [69]. It is widely recognized that the heightened mechanical stress linked to elevated BMI leads to an increase in bone density to support the additional body weight [70]. The connection between BMI and bone density is believed to primarily involve total fat mass. In the general population, an increased total fat mass correlates with elevated sex hormones, which consequently improves BMD [71]. Therefore, in light of these results and the outcome of our research, we also need to focus more on bone health in patients with low BMI among KTRs during their follow-up. We also reported high prevalence of osteoporosis in KTRs with pretransplant diabetes mellitus, which was 29% (95% CI 19–39). Chronic high blood glucose probably causes a buildup of AGEs in bone collagen, which may damage the bone's microarchitecture, resulting in irregular bone metabolism and decreased bone quality [72]. Furthermore, the anti-diabetic drugs rosiglitazone and pioglitazone elevate the risk of primarily non-vertebral fractures [73, 74] probably due to suppression of osteoblast activity and stimulation of osteoclast formation [75]. Additionally, diabetes could trigger types of complications, including peripheral neuropathy and optic neuropathy, which increase the patient's likelihood of falling and the risk of fractures [76].

### Limitations and recommendations for future studies

To the best of our knowledge, this systematic review and meta-analysis is the first to specifically evaluate the prevalence of osteoporosis among KTRs. The interpretation of our findings must take into account several limitations. In the first place, most of the results in this review are based on observational data, which raises concerns about high heterogeneity of the included studies that may result from several factors such as inclusion criteria of the studies including study date, study population, sample size, ethnicity, and gender distribution. We tried to reduce the impact of heterogeneity in our analysis by employing a random-effects model for the meta-analysis, which presumes that the primary studies are diverse and provides a more cautious estimate of effect. We also attempted to address bias through meta-regression and subgroup analyses. Additionally, the majority of the studies in our meta-analysis originate from Asia and Europe, lacking adequate research from Africa, Australia, and to a lesser degree, America; therefore, the limited number of studies in these regions hinders the ability to accurately depict the true prevalence. Therefore, further epidemiological research is recommended to evaluate the prevalence of osteoporosis in the nations throughout these continents. Moreover, the majority of the studies included in the analysis were conducted in countries with high and very high HDI, which may limit the generalizability of the findings to other regions. Therefore, caution should be exercised when extrapolating our findings to other countries and we suggest obtaining more precise and country-specific prevalence rates, particularly in medium and low HDI nations, to improve the precision of budget impact models in estimating medication expenditures, costs of associated medical services (like office visits, inpatient care), and the estimates of required physicians, nurses, and other healthcare professionals and facilities needed to achieve the desired level of care access. Additionally, it is recommended that future research provides more detailed definitions when presenting the prevalence rates, specifically by clarifying the osteoporosis rates in KTRs according to each bone site instead of merely reporting a general osteoporosis rate. Furthermore, our study is affected by language bias and lack of searching for gray literature. Furthermore, in this meta-analysis, data related to study design was not employed, and our analyses lacked standardization across the studies. The application of different designs and methods may have led to undiscovered biases, possibly jeopardizing the credibility of our results. Regarding methodological quality, many studies exhibited low quality, which could affect the validity of overall results and limit the generalizability of the results, even within the studied areas; therefore, we suggest conducting more high-quality research to improve our understanding of osteoporosis prevalence in KTRs. To overcome these limitations, we have employed several strategies, such as utilizing random-effects models in our analysis and performing evaluations based on outcome definitions for each continent and HDI level. Another aspect to consider is that research data from specific countries was released by the same authors at various times, resulting in the unintentional inclusion of comparable osteoporotic cases. Consequently, there is a chance of a minor overestimation of the osteoporosis rate. Additionally, to reach more definitive conclusions, additional research examining how geographical location and ethnicity influence KTRs is warranted, taking into account the differences in outcomes observed in different continents and nations. Moreover, given the limited research assessing the impact of immunosuppressive therapies on BMD in KTRs, additional studies should be carried out in the future regarding their effects, particularly over an extended duration. In addition, it is difficult to make conclusive statements regarding osteoporosis rates because of differences in outcome definitions, statistical modifications, types of BMD measurement devices, and reference data for BMD measurements among various studies. Additionally, socioeconomic factors may have restricted access to DEXA in certain countries, potentially raising the issue of whether osteoporosis underdiagnosed has taken place in KTR patients. Furthermore, the parameters of DEXA devices differ from country to country, resulting in inconsistencies in the values obtained. Finally, taking into account the trim and fill analysis, the prevalence of osteoporosis in KTRs is affected by publication bias; therefore, future systematic reviews and meta-analyses should implement broader searches to tackle this problem.

## Conclusion

To recapitulate, our systematic review and meta-analysis revealed a high global prevalence of osteoporosis, which the rate was highest in bone site rich in cortical content (ultradistal radius region). Based on the specific bone site, Europe exhibited a higher prevalence of osteoporosis in the lumbar and total hip regions, while the osteoporosis rate in the femoral neck region was higher in Asia compared to Europe. Furthermore, countries with very high HDI levels exhibited the highest prevalence rates at each bone site. The current study sought to address the constraints of prior systematic review meta-analyses by conducting a thorough examination of diverse sources and employing various meta-regression and subgroup analyses. This data is essential for informing healthcare planning and policy decisions, providing insight into current trends and future projections and enabling the provision of long-term epidemiological strategies and necessary treatment facilities for individuals with osteoporosis, thereby mitigating the severe risks of fractures and reducing associated mortality rates. Given the significant clinical, economic, and social impact of osteoporosis, accurate prevalence estimates are crucial for informing policy decisions. These decisions affect the identification of individuals needing treatment and their access to fracture risk reduction through drug therapies and ongoing monitoring.

## Supplementary Information

Below is the link to the electronic supplementary material.

Supplementary file1 (DOCX 916 kb)

Supplementary file2 (DOC 203 kb)

## Data Availability

Data is provided within the manuscript or supplementary information files.

## Abbreviations

KTRs: Kidney transplant recipients
HDI: Human developing index
BMD: Bone mineral density
DEXA: Dual-energy X-ray absorptiometry
WHO: World Health Organization
ISCD: International Society for Clinical Densitometry
KT: Kidney transplantation
ESRD: End-stage renal disease
CKD: Chronic kidney disease
UNDP: United Nations Development Programme
GDP: Gross domestic product
REM: Random effect model
BMI: Body mass index
SMD: Standardized mean difference
mTOR Inhibitors: Mammalian target of rapamycin inhibitors
SDI: Sociodemographic index
LH: Luteotropic hormone
PTH: Parathyroid hormone
