# Supplementary material for: Global and regional prevalence of osteoporosis in kidney transplant recipients: a systematic review and meta-analysis
Source: Clin Exp Med. 2025 Jun 20;25(1):214. doi: 10.1007/s10238-025-01716-w (PMC12181100; doi:10.1007/s10238-025-01716-w)
Supplement: Supplementary file 1 — Supplementary file1 (DOCX 916 kb) [file 10238_2025_1716_MOESM1_ESM.docx]

**Online Supplementary file:**

1. List of supplementary file, **page 1.**
2. Search strategies, **pages 2, 3.**
3. **eTable 1.** List of excluded studies at full-text screening stage with brief reasons, **pages 4, 5.**
4. **eTable 2.** Risk of bias assessment of included studies according to the JBI checklist, **pages 6-13.**
5. **eTables 3.** The characteristics of included studies, **pages 14-21.**
6. **eTables 4.** The osteoporosis rate of the included studies according to each bone site, **pages 22-30.**
7. **eTable 5.** Post hoc subgroup findings, **pages 31-44.**
8. **eFigures 1-26, pages 45-70.**
9. **eFigures 27-39, pages 71-83.**
10. Post hoc **efigures 40-43, pages 84-87.**
11. References, **pages 88-95.**
12. **Search syntaxes based on each database:**

**PubMed = 1883 results**

((Renal Transplantation[tiab] OR Kidney Transplantation[tiab] OR Kidney Graft[tiab] OR Renal Graft[tiab] OR kidney transplant recipients[tiab] OR Renal transplant recipients[tiab] OR Kidney recipients[tiab] OR Renal recipients[tiab]) AND (Osteoporoses[tiab] OR Osteoporosis[tiab] OR Bone Loss[tiab] OR bone mineral density[tiab] OR bone disease[tiab] OR bone health[tiab]))

**Web of Science = 3090 results**

((TS=Renal Transplantation OR TS=Kidney Transplantation OR TS=Kidney Graft OR TS=Renal Graft OR TS=kidney transplant recipients OR TS=Renal transplant recipients OR TS=Kidney recipients OR TS=Renal recipients) AND (TS=Osteoporoses OR TS=Osteoporosis OR TS=Bone Loss OR TS=bone mineral density OR TS=bone disease OR TS=bone health))

**Scopus = 4835 results**

((TITLE-ABS(Renal Transplantation) OR TITLE-ABS(Kidney Transplantation) OR TITLE-ABS(Kidney Graft) OR TITLE-ABS(Renal Graft) OR TITLE-ABS(kidney transplant recipients) OR TITLE-ABS(Renal transplant recipients) OR TITLE-ABS(Kidney recipients) OR TITLE-ABS(Renal recipients)) AND (TITLE-ABS(Osteoporoses) OR TITLE-ABS(Osteoporosis) OR TITLE-ABS(Bone Loss) OR TITLE-ABS(bone mineral density) OR TITLE-ABS(bone disease) OR TITLE-ABS(bone health)))

**Science direct = 682 results**

((title-abs-key(Renal Transplantation) OR title-abs-key(Kidney Transplantation) OR title-abs-key(Kidney Graft) OR title-abs-key(Renal Graft) OR title-abs-key(Kidney recipients) OR title-abs-key(Renal recipients) OR title-abs-key(kidney transplant recipients) OR title-abs-key(Renal transplant recipients)) AND (title-abs-key(Osteoporosis) OR title-abs-key(bone loss) OR title-abs-key(Osteoporoses) OR title-abs-key(bone mineral density) OR title-abs-key(bone disease) OR title-abs-key(bone health)))

**Embase = 2558 results**

((“Renal Transplantation”:ti,ab OR “Kidney Transplantation”:ti,ab OR “Renal Graft”:ti,ab OR “Kidney Graft”:ti,ab OR “Renal recipients”:ti,ab OR “Kidney recipients”:ti,ab OR “Renal transplant recipients”:ti,ab OR “kidney transplant recipients”:ti,ab) AND (Osteoporosis:ti,ab OR “bone loss”:ti,ab OR Osteoporoses:ti,ab OR “bone mineral density”:ti,ab OR “bone disease”:ti,ab OR “bone health”:ti,ab))

**Google scholar = 35 results**

allintitle: "Renal transplantation" + osteoporosis

1. **eTable 1.** List of excluded studies at full-text screening stage with brief reasons.

| No. | Author | Title | Brief report for excluded studies |
| --- | --- | --- | --- |
| 1 | Agras et al, 2005 | Relationship between leptin and bone mineral density in renal transplant recipients | Insufficient data reporting |
| 2 | Ahn et al, 2006 | Risk factors for changes in bone mineral density and the effect of antiosteoporosis management after renal transplantation | Insufficient data reporting |
| 3 | Ban et al, 2017 | Clinical effects of pre-transplant serum 25-hydroxyvitamin D level on post-transplant immunologic and non-immunologic outcomes in kidney transplant recipients | Insufficient data reporting |
| 4 | Bergua et al, 2008 | Effect of Cinacalcet on hypercalcemia and bone mineral density in renal transplanted patients with secondary hyperparathyroidism | Insufficient data reporting |
| 5 | Bozkaya et al, 2008 | Impact of Calcineurin Inhibitors on Bone Metabolism in Primary Kidney Transplant Patients | Insufficient data reporting |
| 6 | Brandenburg et al, 2004 | Early rapid loss followed by long-term consolidation characterizes the development of lumbar bone mineral density after kidney transplantation | Insufficient data reporting |
| 7 | Bubenicek et al, 2008 | Early bone mineral density loss after renal transplantation and pre-transplant PTH: A prospective study | Insufficient data reporting |
| 8 | Canoz et al, 2015 | Successful Renal Transplantation, Bone Mineral Densitometry, and Affecting Factors | Insufficient data reporting |
| 9 | Casezet al, 2002 | Changes in bone mineral density over 18 months following kidney transplantation: The respective roles of prednisone and parathyroid hormone | Insufficient data reporting |
| 10 | Chandran et al, 2019 | Addressing bone quality and bone density after renal transplantation: A prospective evaluation of the evolution of trabecular bone score and bone mineral density over the first 5 years following renal transplantation in Asian patients | Insufficient data reporting |
| 11 | Demirci et al, 2018 | Pre-transplant low paratyroid hormone level: A risk factor for post-transplant osteoporosis and arterial stiffness | Insufficient data reporting |
| 12 | Higuchi et al, 2022 | Risk factors for subchondral insufficiency fracture of the femoral head in renal transplant patients | Insufficient data reporting |
| 13 | Hwang et al, 2018 | Changes in Bone Mineral Density After Kidney Transplantation | Insufficient data reporting |
| 14 | Lim et al, 2011 | Prevalence and patterns of bone loss in the first year after renal transplant in South East Asian patients | Insufficient data reporting |
| 15 | Marcén et al, 2006 | Lumbar bone mineral density in renal transplant patients on neoral and tacrolimus:: A four-year prospective study | Insufficient data reporting |
| 16 | Marcen et al, 2004 | Lumbar bone mineral density (BMD) after kidney transplantation.: A prospective study | Insufficient data reporting |
| 17 | Sessa et al, 2010 | Immunosuppressive Agents and Bone Disease in Renal Transplant Patients With Hypercalcemia | Insufficient data reporting |
| 18 | Yu et al, 2014 | Osteoporosis and fractures after solid organ transplantation: A nationwide population-based cohort study | Insufficient data reporting |
| 19 | Albano et al, 2005 | Effects of tacrolimus vs cyclosporin-A on bone metabolism after kidney transplantation: A cross-sectional study in 28 patients | Non-compliance with inclusion criteria |
| 20 | De Alarcon et al, 2010 | Bone mineral density in patients with renal hyperparathyroidism undergoing surgery: relationship with bone parameters | Non-compliance with inclusion criteria |
| 21 | MP et al, 2015 | Assessment of lipid profiles and bone mineral density in renal transplant patients | Non-compliance with inclusion criteria |
| 22 | Karataş et al, 2012 | Risk Factors for Osteoporosis in Renal Transplant Recipients | Non-compliance with inclusion criteria |
| 23 | AR et al, 2015 | Osteoporosis and body mass index in renal transplant recipients | Non-compliance with inclusion criteria |
| 24 | Zhang et al, 2015 | Investigation of bone loss and its related factors in renal transplant recipients | Non-compliance with inclusion criteria |
| 25 | Hassan et al, 2021 | Prevalence of Musculoskeletal Manifestations in Adult Kidney Transplant's Recipients: A Systematic Review | Review article |

1. **eTable 2.** Risk of bias assessment of the included studies according to the JBI checklist.

| **Study/Year** | **Type of study** | **Item 1** | **Item 2** | **Item 3** | **Item 4** | **Item 5** | **Item 6** | **Item 7** | **Item 8** | **Item 9** | **Risk of bias** |
| --- | --- | --- | --- | --- | --- | --- | --- | --- | --- | --- | --- |
| Afifi et al., 2021 | Cohort | 0 | 0 | 0 | 1 | 1 | 1 | 1 | 0 | NA | High |
| Ahmadpoor et al., 2009 | Cross-sectional | 0 | 0 | 0 | 0 | 1 | 1 | 1 | 0 | NA | High |
| Akaberi et al., 2008 | Cross-sectional | 1 | 0 | 1 | 1 | 1 | 1 | 1 | 0 | NA | Medium |
| Akaberi et al., 2006 | Cross-sectional | 1 | 0 | 0 | 0 | 1 | 1 | 1 | 0 | NA | High |
| Alfano et al., 2018 | Case-control | 0 | 0 | 0 | 0 | 1 | 1 | 1 | 0 | NA | High |
| Al-Aradi et al., 2009 | Cohort | 1 | 1 | 1 | 0 | 1 | 1 | 1 | 0 | NA | Medium |
| Alfieri et al., 2021 | Cohort | 0 | 0 | 0 | 1 | 1 | 1 | 1 | 0 | NA | High |
| Aleksova et al., 2017 | Cohort | 0 | 0 | 1 | 1 | 1 | 1 | 1 | 0 | NA | Medium |
| Alis et al., 2017 | Cross-sectional | 1 | 0 | 0 | 0 | 1 | 1 | 1 | 0 | NA | High |
| Atallah et al., 2008 | Cohort | 1 | 1 | 0 | 0 | 1 | 1 | 1 | 0 | NA | Medium |
| Basir et al., 2019 | Cross-sectional | 1 | 0 | 0 | 1 | 1 | 1 | 1 | 0 | NA | Medium |
| Bayat et al., 2007 | Cross-sectional | 1 | 1 | 0 | 0 | 1 | 1 | 1 | 0 | NA | Medium |
| Batteux et al., 2020 | Cohort | 0 | 1 | 1 | 1 | 1 | 1 | 1 | 0 | NA | Medium |
| Batteux et al., 2020b | Cohort | 1 | 0 | 1 | 1 | 1 | 1 | 1 | 0 | NA | Medium |
| Battaglia et al., 2022 | Cohort | 0 | 0 | 0 | 1 | 1 | 1 | 1 | 1 | NA | Medium |
| Battaglia et al., 2023 | Cohort | 0 | 0 | 0 | 1 | 1 | 1 | 1 | 1 | NA | Medium |
| Berczi et al., 2003 | Cohort | 1 | 0 | 0 | 0 | 1 | 1 | 1 | 0 | NA | High |
| Bonani et al., 2016 | RCT | 1 | 0 | 0 | 1 | 1 | 1 | 1 | 0 | NA | Medium |
| Bonani et al., 2019 | RCT | 0 | 0 | 0 | 0 | 1 | 1 | 1 | 0 | NA | High |
| Braga Júnio et al., 2006 | Cross-sectional | 1 | 1 | 1 | 0 | 1 | 1 | 1 | 1 | NA | Low |
| Brandenburg et al., 2002 | Cohort | 1 | 0 | 0 | 0 | 1 | 1 | 1 | 0 | NA | High |
| Brandenburg et al., 2005 | Cohort | 1 | 0 | 0 | 0 | 1 | 1 | 1 | 0 | NA | High |
| Braun et al., 2001 | Cohort | 1 | 0 | 0 | 1 | 1 | 1 | 1 | 0 | NA | Medium |
| Brunova et al., 2018 | Cross-sectional | 0 | 0 | 0 | 0 | 1 | 1 | 1 | 0 | NA | High |
| Cayco et al., 2000 | Cross-sectional | 0 | 0 | 0 | 0 | 1 | 1 | 1 | 0 | NA | High |
| Chen et al., 2016 | Cohort | 1 | 0 | 0 | 1 | 1 | 1 | 1 | 0 | NA | Medium |
| Coskun et al., 2016 | Cross-sectional | 1 | 0 | 0 | 0 | 1 | 1 | 1 | 0 | NA | High |
| Cruz et al., 2002 | Cohort | 0 | 0 | 0 | 0 | 1 | 1 | 1 | 0 | NA | High |
| Demir et al., 2023 | Cross-sectional | 0 | 0 | 0 | 0 | 1 | 1 | 1 | 1 | NA | High |
| Dolgos et al., 2008 | Cross-sectional | 1 | 0 | 0 | 1 | 1 | 1 | 1 | 0 | NA | Medium |
| Durieux et al., 2002 | Cross-sectional | 1 | 0 | 0 | 1 | 1 | 1 | 1 | 0 | NA | Medium |
| El-Agroudy et al., 2003 | prospective controlled, randomized | 0 | 0 | 0 | 0 | 1 | 1 | 1 | 0 | NA | High |
| El-Agroudy et al., 2005 | Prospective randomized | 0 | 0 | 0 | 0 | 1 | 1 | 1 | 0 | NA | High |
| Ergun et al., 2020 | Cross-sectional | 0 | 0 | 0 | 0 | 1 | 1 | 1 | 0 | NA | High |
| Evenepoel et al., 2019 | Cohort | 1 | 1 | 1 | 1 | 1 | 1 | 1 | 1 | NA | Low |
| Evenepoel et al., 2019b | Cohort | 1 | 1 | 1 | 1 | 1 | 1 | 1 | 1 | NA | Low |
| Falkiewicz et al., 2009 | Cohort | 0 | 0 | 0 | 0 | 1 | 1 | 1 | 0 | NA | High |
| Ferreira et al., 2021 | Cross-sectional | 1 | 0 | 0 | 0 | 1 | 1 | 1 | 0 | NA | High |
| Gallego et al., 2006 | Cross-sectional | 1 | 0 | 0 | 0 | 1 | 1 | 1 | 0 | NA | High |
| Gheiasi et al., 2022 | Cross-sectional | 0 | 0 | 0 | 0 | 1 | 1 | 1 | 0 | NA | High |
| Giannini et al., 2001 | Cross-sectional | 1 | 0 | 0 | 0 | 1 | 1 | 1 | 0 | NA | High |
| Giannini et al., 2002 | Cross-sectional | 0 | 0 | 0 | 0 | 1 | 1 | 1 | 0 | NA | High |
| Giannini et al., 2010 | Cross-sectional | 1 | 0 | 0 | 1 | 1 | 1 | 1 | 0 | NA | Medium |
| Govindarajan et al., 2011 | Cohort | 1 | 0 | 0 | 0 | 1 | 1 | 1 | 0 | NA | High |
| Gregorini et al., 2017 | Cohort | 1 | 0 | 1 | 0 | 1 | 1 | 1 | 1 | NA | Medium |
| Gupta et al., 2012 | Cross-sectional | 1 | 0 | 1 | 1 | 1 | 1 | 1 | 0 | NA | Medium |
| Hameed et al., 2018 | Cross-sectional | 0 | 0 | 0 | 1 | 1 | 1 | 1 | 0 | NA | High |
| Hannarici et al., 2022 | Cross-sectional | 0 | 0 | 0 | 1 | 1 | 1 | 1 | 0 | NA | High |
| Hasan et al., 2020 | Case-control | 0 | 0 | 0 | 1 | 1 | 1 | 1 | 0 | NA | High |
| Heaf et al., 2000 | Cross-sectional | 1 | 0 | 0 | 0 | 1 | 1 | 1 | 0 | NA | High |
| Hori et al., 2023 | Cohort | 0 | 0 | 1 | 0 | 1 | 1 | 1 | 1 | NA | Medium |
| Hsu et al., 2012 | Cross-sectional | 1 | 0 | 0 | 1 | 1 | 1 | 1 | 0 | NA | Medium |
| Hsu et al., 2016 | Cross-sectional | 1 | 0 | 0 | 1 | 1 | 1 | 1 | 0 | NA | Medium |
| Huang et al., 2011 | Cohort | 1 | 0 | 0 | 0 | 1 | 1 | 1 | 0 | NA | High |
| Huang et al., 2012 | Case-control | 0 | 1 | 0 | 0 | 1 | 1 | 1 | 1 | NA | Medium |
| Huang et al., 2013 | Case-control | 0 | 1 | 0 | 0 | 1 | 1 | 1 | 1 | NA | Medium |
| January et al., 2020 | Cohort | 1 | 0 | 1 | 1 | 1 | 1 | 1 | 0 | NA | Medium |
| Jeffery et al., 2003 | RCT | 1 | 1 | 1 | 1 | 1 | 1 | 1 | 0 | NA | Low |
| Jerman et al., 2017 | Cohort | 1 | 0 | 1 | 1 | 1 | 1 | 1 | 0 | NA | Medium |
| Jiménez et al., 2016 | Cross-sectional | 1 | 0 | 0 | 1 | 1 | 1 | 1 | 0 | NA | Medium |
| Jørgensen et al., 2015 | Cross-sectional | 1 | 0 | 1 | 1 | 1 | 1 | 1 | 0 | NA | Medium |
| Jørgensen et al., 2021 | Cohort | 1 | 0 | 1 | 1 | 1 | 1 | 1 | 1 | NA | Low |
| Jørgensen et al., 2023 | Cohort | 1 | 0 | 1 | 1 | 1 | 1 | 1 | 0 | NA | Medium |
| Joy et al., 2002 | Cross-sectional | 1 | 0 | 0 | 0 | 1 | 1 | 1 | 0 | NA | High |
| Karataş et al., 2022 | Cohort | 0 | 0 | 0 | 0 | 1 | 1 | 1 | 0 | NA | High |
| Kart-Köseoglu et al., 2003 | Cross-sectional | 0 | 0 | 0 | 1 | 1 | 1 | 1 | 0 | NA | High |
| Kaya et al., 2019 | Cross-sectional | 1 | 0 | 0 | 1 | 1 | 1 | 1 | 0 | NA | Medium |
| Keronen et al., 2019 | Cohort | 1 | 0 | 0 | 1 | 1 | 1 | 1 | 0 | NA | Medium |
| Keronen et al., 2022 | Cohort | 0 | 0 | 0 | 0 | 1 | 1 | 1 | 0 | NA | High |
| Keven et al., 2007 | Cross-sectional | 0 | 0 | 0 | 0 | 1 | 1 | 1 | 0 | NA | High |
| Kinsella et al., 2015 | Cross-sectional | 1 | 0 | 0 | 1 | 1 | 1 | 1 | 0 | NA | Medium |
| Kobel et al., 2019 | Cohort | 0 | 0 | 0 | 0 | 1 | 1 | 1 | 0 | NA | High |
| Kovac et al., 2003 | Cohort | 1 | 0 | 0 | 0 | 1 | 1 | 1 | 0 | NA | High |
| Lee et al., 2019 | Cohort | 1 | 1 | 1 | 0 | 1 | 1 | 1 | 0 | NA | Medium |
| Lee et al., 2014 | Cross-sectional | 0 | 0 | 0 | 1 | 1 | 1 | 1 | 0 | NA | High |
| Lim et al., 2009 | Cohort | 1 | 0 | 0 | 0 | 1 | 1 | 1 | 0 | NA | High |
| Lin et al., 2023 | Cross-sectional | 0 | 0 | 0 | 1 | 1 | 1 | 1 | 0 | NA | High |
| Madeira et al., 2014 | Cross-sectional | 0 | 0 | 0 | 1 | 1 | 1 | 1 | 0 | NA | High |
| Małyszko et al., 2003 | Cross-sectional | 0 | 0 | 0 | 0 | 1 | 1 | 1 | 0 | NA | High |
| Marcén et al., 2007 | Cross-sectional | 1 | 0 | 0 | 0 | 1 | 1 | 1 | 0 | NA | High |
| Marques et al., 2019 | prospective randomized trial | 0 | 0 | 0 | 1 | 1 | 1 | 1 | 0 | NA | High |
| Matei et al., 2021 | Cross-sectional | 1 | 0 | 0 | 1 | 1 | 1 | 1 | 0 | NA | Medium |
| Mazzaferro et al., 2006 | Cross-sectional | 1 | 0 | 0 | 0 | 1 | 1 | 1 | 0 | NA | High |
| Mikuls et al., 2003 | Cohort | 1 | 1 | 0 | 1 | 1 | 1 | 1 | 1 | NA | Low |
| Mondry et al., 2001 | Cross-sectional | 1 | 0 | 0 | 0 | 1 | 1 | 1 | 0 | NA | High |
| Montalban et al., 2003 | Cross-sectional | 1 | 0 | 0 | 0 | 1 | 1 | 1 | 0 | NA | High |
| Naga et al., 2023 | Cross-sectional | 0 | 0 | 0 | 1 | 1 | 1 | 1 | 0 | NA | High |
| Nanmoku et al., 2019 | Case-control | 0 | 0 | 0 | 1 | 1 | 1 | 1 | 0 | NA | High |
| Naylor et al., 2014 | Cohort | 1 | 0 | 1 | 1 | 1 | 1 | 1 | 0 | NA | Medium |
| Naylor et al., 2014b | Cohort | 1 | 0 | 1 | 1 | 1 | 1 | 1 | 0 | NA | Medium |
| Nouri et al., 2008 | Cross-sectional | 1 | 0 | 0 | 0 | 1 | 1 | 1 | 0 | NA | High |
| Nowacka-Cieciura et al., 2006 | Cohort | 0 | 0 | 0 | 0 | 1 | 1 | 1 | 0 | NA | High |
| Opelz et al., 2011 | Cohort | 1 | 1 | 1 | 1 | 1 | 1 | 1 | 0 | NA | Low |
| ve Osteoporoz et al., 2021 | Cross-sectional | 1 | 1 | 0 | 0 | 1 | 1 | 1 | 0 | NA | Medium |
| Otaibi et al., 2021 | Cohort | 0 | 0 | 0 | 1 | 1 | 1 | 1 | 0 | NA | High |
| Ozdem et al., 2015 | Case-control | 0 | 0 | 0 | 0 | 1 | 1 | 1 | 0 | NA | High |
| Ozel et al., 2011 | Cohort | 1 | 0 | 0 | 0 | 1 | 1 | 1 | 0 | NA | High |
| Pajouhi et al., 2005 | Cross-sectional | 1 | 0 | 0 | 0 | 1 | 1 | 1 | 0 | NA | High |
| Park et al., 2017 | Cohort | 1 | 0 | 1 | 1 | 1 | 1 | 1 | 0 | NA | Medium |
| Patel et al., 2001 | Cross-sectional | 1 | 0 | 1 | 1 | 1 | 1 | 1 | 0 | NA | Medium |
| Pereira et al., 2010 | Cohort | 0 | 0 | 0 | 0 | 1 | 1 | 1 | 0 | NA | High |
| Prieto et al., 2009 | Cohort | 0 | 0 | 0 | 0 | 1 | 1 | 1 | 0 | NA | High |
| Rathi et al., 2015 | Cohort | 1 | 0 | 0 | 0 | 1 | 1 | 1 | 0 | NA | High |
| Renau et al., 2002 | Cross-sectional | 0 | 0 | 0 | 0 | 1 | 1 | 1 | 0 | NA | High |
| Roberts et al., 2012 | Cross-sectional | 0 | 1 | 0 | 0 | 1 | 1 | 1 | 0 | NA | High |
| Rocha et al., 2016 | Cohort | 0 | 0 | 0 | 1 | 1 | 1 | 1 | 0 | NA | High |
| Roe et al., 2005 | Cross-sectional | 0 | 0 | 0 | 1 | 1 | 1 | 1 | 0 | NA | High |
| Rojas et al., 2003 | Cohort | 0 | 0 | 0 | 0 | 1 | 1 | 1 | 0 | NA | High |
| Rubello et al., 2005 | Cross-sectional | 0 | 0 | 0 | 0 | 1 | 1 | 1 | 0 | NA | High |
| Savaj et al., 2012 | Cross-sectional | 1 | 0 | 0 | 0 | 1 | 1 | 1 | 0 | NA | High |
| Schreiber et al., 2018 | Cohort | 1 | 0 | 0 | 0 | 1 | 1 | 1 | 0 | NA | High |
| Segaud et al., 2018 | Cohort | 1 | 0 | 1 | 1 | 1 | 1 | 1 | 0 | NA | Medium |
| Sezer et al., 2005 | Cohort | 1 | 0 | 0 | 0 | 1 | 1 | 1 | 0 | NA | High |
| Sikgenc et al., 2010 | Cohort | 1 | 0 | 0 | 0 | 1 | 1 | 1 | 0 | NA | High |
| Sun et al., 2023 | Cohort | 0 | 0 | 0 | 1 | 1 | 1 | 1 | 1 | NA | Medium |
| Smets et al., 2004 | Cohort | 0 | 0 | 0 | 0 | 1 | 1 | 1 | 0 | NA | High |
| Sotomayor et al., 2021 | Cross-sectional | 1 | 0 | 1 | 1 | 1 | 1 | 1 | 0 | NA | Medium |
| Stavroulopoulos et al., 2008 | Cross-sectional | 1 | 0 | 0 | 0 | 1 | 1 | 1 | 0 | NA | High |
| Techawathanawanna et al., 2005 | Cross-sectional | 1 | 0 | 0 | 1 | 1 | 1 | 1 | 0 | NA | Medium |
| Toro et al., 2003 | Cohort | 1 | 0 | 0 | 0 | 1 | 1 | 1 | 0 | NA | High |
| Trabulus et al., 2003 | Cross-sectional | 1 | 0 | 0 | 0 | 1 | 1 | 1 | 0 | NA | High |
| Tsai et al., 2023 | Cohort | 1 | 1 | 1 | 1 | 1 | 1 | 1 | 0 | NA | Low |
| Tsujita et al., 2020 | RCT | 0 | 0 | 0 | 1 | 1 | 1 | 1 | 0 | NA | High |
| Tutal et al., 2013 | Cross-sectional | 1 | 0 | 0 | 0 | 1 | 1 | 1 | 0 | NA | High |
| Ugur et al., 2001 | Cross-sectional | 1 | 0 | 0 | 1 | 1 | 1 | 1 | 0 | NA | Medium |
| Unal et al., 2010 | Cross-sectional | 1 | 0 | 0 | 0 | 1 | 1 | 1 | 0 | NA | High |
| Valencia et al., 2018 | Cross-sectional | 0 | 0 | 0 | 1 | 1 | 1 | 1 | 0 | NA | High |
| Velioglu et al., 2021 | Cohort | 0 | 1 | 0 | 0 | 1 | 1 | 1 | 1 | NA | Medium |
| Walder et al., 2018 | Cross-sectional | 1 | 0 | 0 | 0 | 1 | 1 | 1 | 0 | NA | High |
| Wang et al., 2008 | Cross-sectional | 0 | 0 | 0 | 0 | 1 | 1 | 1 | 0 | NA | High |
| Wang et al., 2021 | Cross-sectional | 0 | 0 | 0 | 1 | 1 | 1 | 1 | 0 | NA | High |
| Wong et al., 2005 | Cohort | 1 | 0 | 0 | 0 | 1 | 1 | 1 | 0 | NA | High |
| Yanishi et al., 2018 | Cross-sectional | 1 | 0 | 0 | 1 | 1 | 1 | 1 | 0 | NA | Medium |
| Yavuz et al., 2022 | Cohort | 0 | 1 | 0 | 0 | 1 | 1 | 1 | 0 | NA | High |
| Yu et al., 2012 | Cohort | 0 | 0 | 0 | 0 | 1 | 1 | 1 | 0 | NA | High |
| Žilinská et al., 2017 | Cohort | 1 | 0 | 0 | 0 | 1 | 1 | 1 | 0 | NA | High |

High risk of bias: score 3 or 4, Medium risk of bias: score 5 or 6, Low risk of bias: score 7 or 8.

**eTable 3. The characteristics of included studies.**

| Author, year | Country | HDI Tier | Study type | Study participants | Male /female | Mean/Median age at the time of study | Osteoporosis definition | Osteoporotic male/female patients |
| --- | --- | --- | --- | --- | --- | --- | --- | --- |
| Kovac et al., 2003[1] | Slovenia | Very high | Cohort | 46 | 25/21 | N/M | T score <-2.5 | N/M |
| Marcén et al., 2007[2] | Spain | Very high | Cross-sectional | 40 | 24/16 | 40-49 | T score <-2.5 | N/M |
| Jørgensen et al. 2016[3] | Norway | Very high | Cross-sectional | 701 | 467/234 | >49 | T-score ≤ -2.5 | N/M |
| Jimenez et al., 2016[4] | Spain | Very high | Cross-sectional | 138 | 79/59 | N/M | T-score ≤ -2.5 | N/M |
| Batteux et al., 2020b[5] | France | Very high | Cohort | 310 | 194/116 | >49 | T-score ≤ –2.5 | N/M |
| Naga et al., 2023[6] | Egypt | high | Cross-sectional | 60 | 60/0 | 40-49 | T-score < -2.5 | N/M |
| Braga Júnior et al., 2006[7] | Brazil | high | Cross-sectional | 191 | 94/97 | 40-49 | T-score ≤ –2.5 | N/M |
| Ahmadpoor et al., 2009[8] | Iran | high | Cross-sectional | 77 | 49/28 | <40 | T-score < -2.5 | N/M |
| Akaberi et al., 2006[9] | Sweden | Very high | Cross-sectional | 41 | 29/12 | N/M | T-score ≤ –2.5 | N/M |
| Akaberi et al., 2008[10] | Sweden | Very high | Cross-sectional | 238 | 146/92 | N/M | T-score ≤ –2.5 | N/M |
| Al-Aradi et al., 2009[11] | Ireland | Very high | Cohort | 774 | 453/321 | >49 | N/S | N/M |
| Alis et al., 2017[12] | Turkey | Very high | Cross-sectional | 109 | 82/27 | <40 | T-score ≤ –2.5 | N/M |
| Atallah et al., 2008[13] | Egypt | high | Cohort | 95 | 73/22 | <40 | T score <-2.5 | N/M |
| Bayat et al., 2007[14] | Iran | high | Cross-sectional | 68 | 47/21 | <40 | N/S | 25/11 |
| Brandenburg et al., 2002[15] | Germany | Very high | Cohort | 26 | 13/13 | 40-49 | T score <-2.5 | N/M |
| Brandenburg et al., 2005[16] | Germany | Very high | Cohort | 67 | 38/29 | 40-49 | T score <-2.5 | N/M |
| Cayco et al., 2000[17] | United States | Very high | Cross-sectional | 69 | 40/29 | 40-49 | T-score ≤ –2.5 | N/M |
| Dolgos et al., 2008[18] | Norway | Very high | Cross-sectional | 133 | 91/42 | >49 | T-score ≤ –2.5 | N/M |
| Durieux et al., 2002[19] | France | Very high | Cross-sectional | 59 | 32/27 | 40-49 | T score <-2.5 | N/M |
| Evenepoel et al., 2019[20] | Belgium | Very high | Cohort | 502 | 305/197 | >49 | T-score ≤ –2.5 | 57/54 |
| Evenepoel et al., 2019b[21] | Belgium | Very high | Cohort | 468 | 284/184 | >49 | T-score ≤ –2.5 | N/M |
| Falkiewicz et al., 2009[22] | Poland | Very high | Cohort | 90 | 53/37 | 40-49 | N/S | N/M |
| Ferreira et al., 2021[23] | Portugal | Very high | Cross-sectional | 67 | 46/21 | N/M | T-score ≤ –2.5 | N/M |
| Gallego et al., 2006[24] | Spain | Very high | Cross-sectional | 106 | 54/52 | >49 | T-score ≤ –2.5 | N/M |
| Gregorini et al., 2017[25] | Italy | Very high | Cohort | 297 | 194/103 | >49 | T score <-2.5 | N/M |
| Hsu et al., 2012[26] | Taiwan | high | Cross-sectional | 65 | 41/24 | >49 | T-score ≤ –2.5 | N/M |
| Hsu et al., 2016[27] | Taiwan | high | Cross-sectional | 69 | 43/26 | >49 | T-score ≤ –2.5 | N/M |
| Huang et al., 2011[28] | Taiwan | high | Cohort | 84 | 40/44 | >49 | T score <-2.5 | 20/28 |
| Gheiasi et al., 2022[29] | Iran | high | Cross-sectional | 69 | 47/22 | 40-49 | T-score ≤ –2.5 | N/M |
| Ozel et al., 2011[30] | Turkey | Very high | Cohort | 44 | 29/15 | <40 | T score <-2.5 | 2/3 |
| Joy et al., 2002[31] | United States | Very high | Cross-sectional | 31 | 11/20 | >49 | T score <-2.5 | 4/3 |
| Kaya et al., 2019[32] | Turkey | Very high | Cross-sectional | 117 | 86/31 | <40 | T score <-2.5 | N/M |
| Alfieri et al., 2021[33] | Italy | Very high | Cohort | 32 | 11/21 | >49 | T score <-2.5 | N/M |
| Wang et al., 2021[34] | China | high | Cross-sectional | 216 | 156/60 | 40-49 | T-score ≤ –2.5 | N/M |
| Mikuls et al., 2003[35] | United States | Very high | Cohort | 45 | 26/19 | 40-49 | T-score ≤ –2.5 | N/M |
| Battaglia et al., 2022[36] | Italy | Very high | Cohort | 100 | 69/31 | >49 | T-score ≤ –2.5 | 19/11 |
| Battaglia et al., 2023[37] | Italy | Very high | Cohort | 69 | 51/18 | >49 | T-score ≤ –2.5 | N/M |
| Mondry et al., 2001[38] | Germany | Very high | Cross-sectional | 50 | 21/29 | 40-49 | T score <-2.5 | N/M |
| Pajouhi et al., 2005[39] | Iran | high | Cross-sectional | 42 | 20/22 | 40-49 | T-score ≤ –2.5 | N/M |
| Lee et al., 2019[40] | South Korea | Very high | Cohort | 941 | 587/354 | 40-49 | T-score ≤ –2.5 | N/M |
| Lee et al., 2014[41] | Taiwan | high | Cross-sectional | 69 | 43/26 | >49 | T score <-2.5 | N/M |
| Opelz et al., 2011[42] | Germany | Very high | Cohort | 20509 | 12628/7881 | 40-49 | N/S | N/M |
| Park et al., 2017[43] | South Korea | Very high | Cohort | 207 | 111/96 | N/M | T-score ≤ –2.5 | N/M |
| Patel et al., 2001[44] | United Kingdom | Very high | Cross-sectional | 165 | 95/70 | 40-49 | T-score ≤ –2.5 | N/M |
| Velioglu et al., 2021[45] | Turkey | Very high | Cohort | 153 | 76/77 | 40-49 | T-score ≤ –2.5 | 5/7 |
| Keronen et al., 2019[46] | Finland | Very high | Cohort | 27 | 22/5 | 40-49 | T score <-2.5 | N/M |
| Keronen et al., 2022[47] | Finland | Very high | Cohort | 109 | 65/44 | >49 | T-score ≤ –2.5 | N/M |
| Renau et al., 2002[48] | Spain | Very high | Cross-sectional | 160 | 87/73 | >49 | T-score ≤ –2.5 | N/M |
| Roe et al., 2004[49] | United Kingdom | Very high | Cross-sectional | 134 | 134/0 | 40-49 | T score <-2.5 | N/M |
| Rojas et al., 2003[50] | Venezuela | Medium | Cohort | 20 | 12/8 | <40 | Bone histology | N/M |
| Segaud et al., 2018[51] | France | Very high | Cohort | 259 | 163/96 | >49 | T-score ≤ –2.5 | N/M |
| Sezer et al., 2005[52] | Turkey | Very high | Cohort | 82 | 55/27 | <40 | T-score ≤ –2.5 | N/M |
| Sikgenc et al., 2010[53] | Turkey | Very high | Cohort | 85 | 57/28 | <40 | T score <-2.5 | N/M |
| Lin et al., 2023[54] | Taiwan | high | Cross-sectional | 66 | 41/25 | >49 | T-score ≤ –2.5 | 2/6 |
| Toro et al., 2003[55] | Spain | Very high | Cohort | 123 | 51/72 | 40-49 | T-score ≤ –2.5 | 4/26 |
| Ugur et al., 2001[56] | Turkey | Very high | Cross-sectional | 130 | 81/49 | <40 | T score <-2.5 | N/M |
| Unal et al., 2010[57] | Turkey | Very high | Cross-sectional | 70 | 50/20 | <40 | T score <-2.5 | 19/11 |
| Wang et al., 2008[58] | Taiwan | high | Cross-sectional | 67 | N/M | N/M | T score <-2.5 | N/M |
| Wong et al., 2005[59] | China | high | Cohort | 31 | 19/12 | N/M | T score <-2.5 | N/M |
| Aleksova et al., 2017[60] | Australia | Very high | Cohort | 146 | 75/71 | >49 | T-score ≤ –2.5 | N/M |
| Trabulus et al., 2003[61] | Turkey | Very high | Cross-sectional | 65 | 43/22 | N/M | T score <-2.5 | N/M |
| Otaibi et al., 2021[62] | Saudi Arabia | Very high | Cohort | 53 | N/M | N/M | T score <-2.5 | 19/20 |
| Valencia et al., 2018[63] | Colombia | high | Cross-sectional | 56 | 29/27 | 40-49 | T-score ≤ –2.5 | N/M |
| Karataş et al., 2022[64] | Turkey | Very high | Cohort | 85 | 59/26 | 40-49 | T-score ≤ –2.5 | N/M |
| Jørgensen et al., 2023 [65] | Belgium | Very high | Cohort | 209 | 136/73 | >49 | T score <-2.5 | N/M |
|  |  |  |  | 207 | N/M | N/M | T score <-2.5 | N/M |
|  |  |  |  | 124 | N/M | N/M | T score <-2.5 | N/M |
| Sotomayor et al., 2020[66] | Netherlands | Very high | Cross-sectional | 678 | 394/284 | >49 | T-score ≤ –2.5 | 24/41 |
| Govindarajan et al., 2011[67] | India | Medium | Cohort | 56 | 47/9 | <40 | T-score ≤ –2.5 | N/M |
| Yavuz et al., 2022[68] | Turkey | Very high | Cohort | 264 | 140/124 | 40-49 | T-score ≤ –2.5 | N/M |
| Jørgensen et al., 2021[69] | Belgium | Very high | Cohort | 108 | N/M | N/M | T score <-2.5 | N/M |
|  |  |  |  | 106 | N/M | N/M | T score <-2.5 | N/M |
|  |  |  |  | 91 | N/M | N/M | T score <-2.5 | N/M |
| Sun et al., 2023[70] | China | high | Cohort | 95 | 68/27 | 40-49 | T-score ≤ –2.5 | N/M |
| ve Osteoporoz et al., 2021[71] | Turkey | Very high | Cross-sectional | 130 | 90/40 | 40-49 | T score <-2.5 | 16/8 |
| Techawathanawanna et al., 2005[72] | Thailand | Very high | Cross-sectional | 102 | 52/50 | 40-49 | T score <-2.5 | N/M |
| Savaj et al., 2012[73] | Iran | high | Cross-sectional | 113 | 55/58 | 40-49 | T score <-2.5 | N/M |
| Afifi et al., 2021[74] | Kuwait | Very high | Cohort | 100 | 49/51 | N/M | T-score ≤ –2.5 | N/M |
| Berczi et al., 2003[75] | Hungary | Very high | Cohort | 240 | 150/90 | N/M | T score <-2.5 | 10/6 |
| Braun et al., 2001[76] | United States | Very high | Cohort | 89 | N/M | >49 | N/S | N/M |
| Brunova et al., 2018[77] | Czechia | Very high | Cross-sectional | 34 | N/M | N/M | T-score ≤ –2.5 | N/M |
| Chen et al., 2016[78] | Sweden | Very high | Cohort | 66 | 45/21 | 40-49 | T-score ≤ –2.5 | N/M |
| Cruz et al., 2002[79] | United States | Very high | Cohort | 58 | 39/19 | 40-49 | T-score ≤ –2.5 | N/M |
| El-Agroudy et al., 2003[80] | Egypt | high | prospective controlled, randomized | 40 | 40/0 | N/M | N/S | N/M |
| El-Agroudy et al., 2005[81] | Egypt | high | prospective randomized | 60 | 60/0 | N/M | N/S | N/M |
| Bonani et al., 2016[82] | Switzerland | Very high | RCT | 90 | 57/33 | N/M | T score <-4 | N/M |
| Giannini et al., 2001[83] | Italy | Very high | Cross-sectional | 40 | 27/13 | 40-49 | T score <-2.5 | N/M |
| Giannini et al., 2002[84] | Italy | Very high | Cross-sectional | 69 | 47/22 | 40-49 | T score <-2.5 | N/M |
| Giannini et al., 2010[85] | Italy | Very high | Cross-sectional | 125 | 87/38 | >49 | T-score ≤ –2.5 | N/M |
| Ergun et al., 2020[86] | Turkey | Very high | Cross-sectional | 69 | 52/17 | 40-49 | T-score ≤ –2.5 | N/M |
| Jeffery et al., 2003[87] | Canada | Very high | RCT | 211 | 149/62 | N/M | T score <-2.5 | N/M |
| Kart-Köseoglu et al., 2003[88] | Turkey | Very high | Cross-sectional | 82 | 56/26 | <40 | T-score ≤ –2.5 | N/M |
| Kinsella et al., 2015[89] | Ireland | Very high | Cross-sectional | 64 | 39/25 | 40-49 | T-score ≤ –2.5 | N/M |
| Kobel et al., 2019[90] | Switzerland | Very high | Cohort | 54 | 31/23 | >49 | N/S | N/M |
| Lim et al., 2009[91] | Australia | Very high | Cohort | 97 | 54/43 | >49 | T score <-2.5 | 6/10 |
| Małyszko et al., 2003[92] | Poland | Very high | Cross-sectional | 26 | 18/8 | N/M | T score <-2.5 | N/M |
| Marques et al., 2019[93] | Brazil | high | prospective randomized trial | 32 | 19/13 | N/M | T-score ≤ –2.5 | N/M |
| Mazzaferro et al., 2006[94] | Italy | Very high | Cross-sectional | 53 | 31/22 | 40-49 | T score <-2.5 | N/M |
| Nanmoku et al., 2019[95] | Japan | Very high | Case-control | 14 | 5/9 | 40-49 | T-score ≤ –2.5 | N/M |
| Naylor et al., 2014[96] | Canada | Very high | Cohort | 458 | 291/167 | 40-49 | T-score ≤ –2.5 | N/M |
| Naylor et al., 2014b[97] | Canada | Very high | Cohort | 326 | 199/127 | 40-49 | N/S | N/M |
| Nowacka-Cieciura et al., 2006[98] | Poland | Very high | Cohort | 66 | N/M | N/M | N/S | N/M |
| Hameed et al., 2018[99] | Iraq | Medium | Cross-sectional | 70 | 37/33 | 40-49 | T score <-2.5 | N/M |
| Rathi et al., 2015[100] | India | Medium | Cohort | 75 | 58/17 | <40 | T score <-2.5 | N/M |
| Roberts et al., 2012[101] | Trinidad and Tobago | Very high | Cross-sectional | 73 | 38/35 | <40 | N/S | N/M |
| Rubello et al., 2005[102] | Italy | Very high | Cross-sectional | 75 | 50/25 | 40-49 | T score <-2.5 | N/M |
| Schreiber et al., 2018[103] | Switzerland | Very high | Cohort | 70 | 42/28 | >49 | T score <-2.5 | N/M |
| Madeira et al., 2014[104] | Brazil | high | Cross-sectional | 88 | 52/36 | 40-49 | T-score ≤ –2.5 | N/M |
| Yu et al., 2012[105] | Australia | Very high | Cohort | 30 | 17/13 | >49 | T score <-2.5 | N/M |
| Žilinská et al., 2017[106] | Slovakia | Very high | Cohort | 88 | 56/32 | N/M | N/S | N/M |
| Tsai et al., 2023[107] | Taiwan | high | Cohort | 5309 | 2873/2436 | N/M | N/S | N/M |
| Hori et al., 2023[108] | Japan | Very high | Cohort | 342 | 220/122 | >49 | T score <-2.5 | N/M |
| Hannarici et al., 2022[109] | Turkey | Very high | Cross-sectional | 118 | 83/35 | 40-49 | T-score ≤ –2.5 | N/M |
| Gupta et al., 2012[110] | Canada | Very high | Cross-sectional | 389 | 247/142 | N/M | T score <-2.5 | 18/9 |
| Basir et al., 2019[111] | Turkey | Very high | Cross-sectional | 78 | 55/23 | 40-49 | T-score ≤ –2.5 | N/M |
| Demir et al., 2023[112] | Turkey | Very high | Cross-sectional | 26 | 20/6 | 40-49 | T-score ≤ -2.5 | N/M |
| Batteux et al., 2020[113] | France | Very high | Cohort | 356 | 226/130 | >49 | T-score ≤ -2.5 | N/M |
| Coskun et al., 2016[114] | Turkey | Very high | Cross-sectional | 106 | 76/30 | 40-49 | T score <-2.5 | N/M |
| Heaf et al., 2000[115] | Denmark | Very high | Cross-sectional | 125 | 70/55 | 40-49 | T score <-2.5 | N/M |
| Keven et al., 2007[116] | Turkey | Very high | Cross-sectional | 68 | N/M | N/M | T-score ≤ –2.5 | N/M |
| Montalban et al., 2003[117] | Spain | Very high | Cross-sectional | 29 | 18/11 | >49 | T score <-2.5 | N/M |
| Ozdem et al., 2015[118] | Turkey | Very high | Case-control | 25 | 17/8 | 40-49 | T score <-2.5 | N/M |
| Stavroulopoulos et al., 2007[119] | United Kingdom | Very high | Cross-sectional | 244 | 151/93 | 40-49 | T-score ≤ –2.5 | N/M |
| Tutal et al., 2013[120] | Turkey | Very high | Cross-sectional | 103 | 69/34 | <40 | T-score ≤ –2.5 | N/M |
| Matei et al., 2021[121] | Romania | Very high | Cross-sectional | 59 | 30/29 | 40-49 | T-score ≤ −2.5 | N/M |
| Nouri et al., 2008[122] | Iran | high | Cross-sectional | 61 | 43/18 | <40 | T score <-2.5 | N/M |
| Bonani et al., 2019[123] | Switzerland | Very high | RCT | 44 | 28/16 | >49 | N/S | N/M |
| January et al., 2020[124] | United States | Very high | Cohort | 1287 | 794/493 | N/M | N/S | N/M |
| Prieto et al., 2009[125] | Spain | Very high | Cohort | 27 | 18/9 | >49 | N/S | N/M |
| Tsujita et al., 2020[126] | Japan | Very high | RCT | 187 | 129/58 | N/M | N/S | N/M |
| Hasan et al., 2020[127] | Iraq | Medium | Case-control | 75 | 52/23 | 40-49 | T-score ≤ –2.5 | N/M |
| Alfano et al., 2018[128] | Italy | Very high | Case-control | 19 | 13/6 | >49 | N/S | N/M |
| Huang et al., 2012[129] | Taiwan | high | Case-control | 76 | 36/40 | N/M | T score <-2.5 | N/M |
| Huang et al., 2013[130] | Taiwan | high | Case-control | 76 | 36/40 | N/M | T score <-2.5 | N/M |
| Jerman et al., 2017[131] | Slovenia | Very high | Cohort | 507 | 280/227 | >49 | T score <-2.5 | N/M |
| Pereira et al., 2010[132] | Portugal | Very high | Cohort | 57 | 20/37 | N/M | T-score ≤ –2.5 | N/M |
| Rocha et al., 2016[133] | Portugal | Very high | Cohort | 48 | 20/28 | N/M | T-score ≤ –2.5 | 17/19 |
| Smets et al., 2004[134] | Netherlands | Very high | Cohort | 19 | 13/6 | N/M | T score <-2.5 | N/M |
| Walder et al., 2018[135] | Switzerland | Very high | Cross-sectional | 40 | 31/9 | 20>49 | T-score ≤ –2.5 | N/M |
| Yanishi et al., 2018[136] | Japan | Very high | Cross-sectional | 58 | 42/16 | 40-49 | T-score ≤ –2.5 | N/M |

HDI Human Development Index, N/M not mentioned, N/S not specified, RCT randomized controlled trial

**eTable 4. The osteoporosis rate of the included studies according to the each bone site.**

| Author | No. General OP | No. Lumbar OP | NO. Femoral neck OP | No. Total hip OP | No. forearm OP | No. ultradistal radius OP |
| --- | --- | --- | --- | --- | --- | --- |
| Kovac et al., 2003[1] | N/M | 14 | N/M | N/M | N/M | N/M |
| Marcén et al., 2007[2] | N/M | 11 | 6 | N/M | N/M | N/M |
| Jørgensen et al. 2016[3] | N/M | 115 | 176 | N/M | N/M | N/M |
| Jimenez et al., 2016[4] | N/M | 32 | 20 | N/M | N/M | N/M |
| Batteux et al., 2020[5] | N/M | 19 | N/M | N/M | N/M | N/M |
| Naga et al., 2023[6] | N/M | 9 | N/M | 3 | 21 | N/M |
| Braga Júnior et al., 2006[7] | N/M | 22 | 21 | N/M | N/M | N/M |
| Ahmadpoor et al.,[8] | 20 | 6 | 19 | N/M | N/M | N/M |
| Akaberi et al., 2006[9] | 8 | N/M | N/M | N/M | N/M | N/M |
| Akaberi et al., 2008[10] | N/M | 34 | N/M | 33 | N/M | N/M |
| Al-Aradi et al., 2009[11] | 115 | N/M | N/M | N/M | N/M | N/M |
| Alis et al., 2017[12] | 24 | 24 | 17 | N/M | N/M | N/M |
| Atallah et al., 2008[13] | 24 | N/M | N/M | N/M | N/M | N/M |
| Bayat et al., 2007[14] | N/M | 6 | 38 | N/M | N/M | N/M |
| Brandenburg et al., 2002[15] | 4 | N/M | N/M | N/M | N/M | N/M |
| Brandenburg et al., 2005[16] | N/M | 15 | N/M | N/M | N/M | N/M |
| Cayco et al., 2000[17] | N/M | 10 | 29 | 12 | N/M | N/M |
| Dolgos et al., 2008[18] | N/M | 16 | N/M | 19 | N/M | N/M |
| Durieux et al., 2002[19] | 31 | 22 | 22 | N/M | N/M | N/M |
| Evenepoel et al., 2019[20] | N/M | 120 | 111 | 54 | N/M | 174 |
| Evenepoel et al., 2019b[21] | N/M | 100 | 65 | N/M | N/M | N/M |
| Falkiewicz et al., 2009[22] | 6 | 8 | 16 | N/M | N/M | N/M |
| Ferreira et al., 2021[23] | 18 | N/M | N/M | N/M | N/M | N/M |
| Gallego et al., 2006[24] | 21 | 21 | 21 | N/M | N/M | N/M |
| Gregorini et al., 2017[25] | 45 | N/M | N/M | N/M | N/M | N/M |
| Hsu et al., 2012[26] | N/M | 6 | N/M | N/M | N/M | N/M |
| Hsu et al., 2016[27] | N/M | 8 | N/M | N/M | N/M | N/M |
| Huang et al., 2011[28] | 48 | N/M | N/M | N/M | N/M | N/M |
| Gheiasi et al., 2022[29] | N/M | 22 | 26 | N/M | N/M | N/M |
| Ozel et al., 2011 [30] | 5 | N/M | N/M | N/M | N/M | N/M |
| Joy et al., 2002[31] | 7 | N/M | N/M | N/M | N/M | N/M |
| Kaya et al., 2019[32] | 41 | 27 | 10 | N/M | N/M | N/M |
| Alfieri et al., 2021[33] | N/M | 23 | 25 | N/M | N/M | N/M |
| Wang et al., 2021[34] | 9 | N/M | N/M | N/M | N/M | N/M |
| Mikuls et al., 2003[35] | N/M | 13 | 4 | N/M | N/M | N/M |
| Battaglia et al., 2022[36] | 30 | 18 | 12 | N/M | N/M | N/M |
| Battaglia et al., 2023[37] | N/M | 15 | 10 | N/M | N/M | N/M |
| Mondry et al., 2001[38] | N/M | N/M | 11 | N/M | N/M | N/M |
| Pajouhi et al., 2005[39] | 8 | 4 | 4 | 4 | N/M | N/M |
| Lee et al., 2019[40] | 104 | N/M | N/M | N/M | N/M | N/M |
| Lee et al., 2014[41] | 8 | N/M | N/M | N/M | N/M | N/M |
| Opelz et al., 2011[42] | 1675 | N/M | N/M | N/M | N/M | N/M |
| Park et al., 2017[43] | N/M | N/M | 98 | N/M | N/M | N/M |
| Patel et al., 2001[44] | N/M | 13 | 17 | N/M | N/M | 53 |
| Velioglu et al., 2021[45] | N/M | 11 | 12 | 12 | N/M | N/M |
| Keronen et al., 2019[46] | N/M | N/M | 6 | N/M | N/M | N/M |
| Keronen et al., 2022[47] | N/M | 9 | 15 | N/M | N/M | N/M |
| Renau et al., 2002[48] | N/M | 61 | 90 | N/M | N/M | N/M |
| Roe et al., 2004[49] | 55 | 23 | 32 | N/M | N/M | 31 |
| Rojas et al., 2003[50] | 2 | N/M | N/M | N/M | N/M | N/M |
| Segaud et al., 2018[51] | 106 | N/M | N/M | N/M | N/M | N/M |
| Sezer et al., 2005[52] | 39 | N/M | N/M | N/M | N/M | N/M |
| Sikgenc et al., 2010[53] | N/M | 25 | 10 | N/M | N/M | N/M |
| Lin et al., 2023[54] | N/M | 8 | N/M | N/M | N/M | N/M |
| Toro et al., 2003[55] | N/M | 18 | 23 | N/M | N/M | N/M |
| Ugur et al., 2001[56] | N/M | 63 | 40 | N/M | N/M | N/M |
| Unal et al., 2010[57] | N/M | N/M | 30 | N/M | N/M | N/M |
| Wang et al., 2008[58] | 31 | N/M | N/M | N/M | N/M | N/M |
| Wong et al., 2005[59] | N/M | 2 | 1 | 1 | N/M | N/M |
| Aleksova et al., 2017[60] | N/M | 15 | 26 | N/M | N/M | N/M |
| Trabulus et al., 2003[61] | 22 | N/M | N/M | N/M | N/M | N/M |
| Otaibi et al., 2021[62] | 22 | N/M | N/M | N/M | N/M | N/M |
| Valencia et al., 2018[63] | 7 | N/M | N/M | N/M | N/M | N/M |
| Karataş et al., 2022[64] | 24 | N/M | N/M | N/M | N/M | N/M |
| Jørgensen et al., 2023[65] | N/M | 44 | N/M | N/M | N/M | N/M |
|  | N/M | N/M | 38 | 25 | N/M | N/M |
|  | N/M | N/M | N/M | N/M | N/M | 50 |
| Sotomayor et al., 2020[66] | 65 | N/M | N/M | N/M | N/M | N/M |
| Govindarajan et al., 2011[67] | 7 | N/M | N/M | N/M | N/M | N/M |
| Yavuz et al., 2022[68] | 53 | N/M | N/M | N/M | N/M | N/M |
| Jørgensen et al., 2021[69] | N/M | 24 | N/M | N/M | N/M | N/M |
|  | N/M | N/M | 27 | 16 | N/M | N/M |
|  | N/M | N/M | N/M | N/M | N/M | 42 |
| Sun et al., 2023[70] | N/M | 4 | 3 | N/M | N/M | N/M |
| ve Osteoporoz et al., 2021[71] | 24 | N/M | N/M | N/M | N/M | N/M |
| Techawathanawanna et al., 2005[72] | 27 | 25 | N/M | 10 | N/M | N/M |
| Savaj et al., 2012[73] | N/M | 14 | 51 | N/M | N/M | N/M |
| Afifi et al., 2021[74] | 23 | N/M | N/M | N/M | N/M | N/M |
| Berczi et al., 2003[75] | 16 | N/M | N/M | N/M | N/M | N/M |
| Braun et al., 2001[76] | 35 | N/M | N/M | N/M | N/M | N/M |
| Brunova et al., 2018[77] | N/M | 28 | N/M | N/M | 24 | N/M |
| Chen et al., 2016[78] | 4 | N/M | N/M | N/M | N/M | N/M |
| Cruz et al., 2002[79] | 27 | N/M | N/M | N/M | N/M | N/M |
| El-Agroudy et al., 2003[80] | N/M | 4 | 6 | N/M | 2 | N/M |
| El-Agroudy et al., 2005[81] | N/M | 7 | 8 | N/M | 3 | N/M |
| Bonani et al., 2016[82] | 9 | N/M | N/M | N/M | N/M | N/M |
| Giannini et al., 2001[83] | N/M | 18 | N/M | 8 | N/M | N/M |
| Giannini et al., 2002[84] | N/M | 25 | N/M | 38 | N/M | N/M |
| Giannini et al., 2010[85] | N/M | 33 | 20 | N/M | N/M | N/M |
| Ergun et al., 2020[86] | N/M | 12 | N/M | 7 | N/M | N/M |
| Jeffery et al., 2003[87] | N/M | 36 | N/M | 32 | N/M | N/M |
| Kart-Köseoglu et al., 2003[88] | N/M | N/M | N/M | 16 | 29 | N/M |
| Kinsella et al., 2015[89] | N/M | 6 | 9 | N/M | N/M | N/M |
| Kobel et al., 2019[90] | 9 | N/M | N/M | N/M | N/M | N/M |
| Lim et al., 2009[91] | 16 | N/M | N/M | N/M | N/M | N/M |
| Małyszko et al., 2003[92] | 9 | N/M | N/M | N/M | N/M | N/M |
| Marques et al., 2019[93] | 12 | N/M | N/M | N/M | N/M | N/M |
| Mazzaferro et al., 2006[94] | 13 | N/M | N/M | N/M | N/M | N/M |
| Nanmoku et al., 2019[95] | 7 | N/M | N/M | N/M | N/M | N/M |
| Naylor et al., 2014[96] | 74 | N/M | N/M | N/M | N/M | N/M |
| Naylor et al., 2014[97] | 14 | N/M | N/M | N/M | N/M | N/M |
| Nowacka-Cieciura et al., 2006[98] | 21 | N/M | N/M | N/M | N/M | N/M |
| Hameed et al., 2018[99] | 37 | N/M | N/M | N/M | N/M | N/M |
| Rathi et al., 2015[100] | 6 | N/M | N/M | N/M | N/M | N/M |
| Roberts et al., 2012[101] | 1 | N/M | N/M | N/M | N/M | N/M |
| Rubello et al., 2005[102] | N/M | 27 | N/M | 42 | N/M | N/M |
| Schreiber et al., 2018[103] | 9 | N/M | N/M | N/M | N/M | N/M |
| Madeira et al., 2014[104] | 25 | N/M | N/M | N/M | N/M | N/M |
| Yu et al., 2012[105] | N/M | 2 | N/M | 3 | N/M | N/M |
| Žilinská et al., 2017[106] | 36 | N/M | N/M | N/M | N/M | N/M |
| Tsai et al., 2023[107] | 103 | N/M | N/M | N/M | N/M | N/M |
| Hori et al., 2023[108] | 93 | N/M | N/M | N/M | N/M | N/M |
| Hannarici et al., 2022[109] | 33 | N/M | N/M | N/M | N/M | N/M |
| Gupta et al., 2012[110] | N/M | 27 | 27 | 24 | N/M | N/M |
| Basir et al., 2019[111] | 34 | 30 | 16 | N/M | N/M | N/M |
| Demir et al., 2023[112] | 3 | N/M | N/M | N/M | N/M | N/M |
| Batteux et al., 2020[113] | 46 | N/M | N/M | N/M | N/M | N/M |
| Coskun et al., 2016[114] | N/M | 29 | 11 | N/M | N/M | N/M |
| Heaf et al., 2000[115] | N/M | 37 | 44 | N/M | N/M | N/M |
| Keven et al., 2007[116] | 22 | N/M | N/M | N/M | N/M | N/M |
| Montalban et al., 2003[117] | N/M | 7 | 13 | N/M | N/M | N/M |
| Ozdem et al., 2015[118] | N/M | N/M | N/M | 3 | N/M | N/M |
| Stavroulopoulos et al., 2007[119] | 64 | 37 | 44 | N/M | N/M | N/M |
| Tutal et al., 2013[120] | 42 | N/M | N/M | N/M | N/M | N/M |
| Matei et al., 2021[121] | 23 | N/M | N/M | N/M | N/M | N/M |
| Nouri et al., 2008[122] | N/M | 13 | 6 | N/M | N/M | N/M |
| Bonani et al., 2019[123] | 4 | N/M | N/M | N/M | N/M | N/M |
| January et al., 2020[124] | 56 | N/M | N/M | N/M | N/M | N/M |
| Prieto et al., 2009[125] | N/M | 4 | 1 | N/M | N/M | N/M |
| Tsujita et al., 2020[126] | 21 | N/M | N/M | N/M | N/M | N/M |
| Hasan et al., 2020[127] | N/M | 25 | 45 | N/M | N/M | N/M |
| Alfano et al., 2018[128] | 2 | N/M | N/M | N/M | N/M | N/M |
| Huang et al., 2012[129] | 41 | N/M | N/M | N/M | N/M | N/M |
| Huang et al., 2013[130] | 30 | N/M | N/M | N/M | N/M | N/M |
| Jerman et al., 2017[131] | 135 | N/M | N/M | N/M | N/M | N/M |
| Pereira et al., 2010[132] | 16 | N/M | N/M | N/M | N/M | N/M |
| Rocha et al., 2016[133] | 36 | 17 | 19 | N/M | N/M | N/M |
| Smets et al., 2004[134] | N/M | 1 | 9 | N/M | N/M | N/M |
| Walder et al., 2018[135] | N/M | N/M | N/M | N/M | N/M | 10 |
| Yanishi et al., 2018[136] | N/M | 10 | N/M | N/M | N/M | N/M |

OP : osteoporosis, N/M: not mentioned

**eTable 5. Post hoc subgroup data.**

| Author, year | Bone site, (type of donor: [Event/Total]) | Comorbidities (Event/Total) | Immunosuppressive treatments (Event/Total) | Simultaneous transplantation (Event/Total) | Age of osteoporotic patients, N (mean±SD) vs. normal BMD, N (mean±SD) | BMI of osteoporotic patients, N (mean±SD) vs. normal BMD, N (mean±SD) | Time since transplantation (months) of osteoporotic patients, N (mean±SD) vs. normal BMD, N (mean±SD) | Duration of hemodialysis – pre-transplant (months) of osteoporotic patients, N (mean±SD) vs. normal BMD, N (mean±SD) |
| --- | --- | --- | --- | --- | --- | --- | --- | --- |
| Kovac et al., 2003[1] | N/M | N/M | N/M | N/M | N/M | N/M | N/M | N/M |
| Marcén et al., 2007[2] | N/M | N/M | N/M | N/M | N/M | N/M | N/M | N/M |
| Jørgensen et al. 2016[3] | N/M | N/M | N/M | N/M | N/M | N/M | N/M | N/M |
| Jimenez et al., 2016[4] | Lumbar (Deceased:32/138)  Femoral neck (Deceased: 20/138) | N/M | N/M | N/M | N/M | N/M | N/M | N/M |
| Batteux et al., 2020[5] | N/M | N/M | N/M | N/M | N/M | N/M | N/M | N/M |
| Naga et al., 2023[6] | Forearm (Living: 21/60) | N/M | N/M | N/M | N/M | N/M | N/M | N/M |
| Braga Júnior et al., 2006[7] | N/M | N/M | N/M | N/M | N/M | N/M | N/M | N/M |
| Ahmadpoor et al.,[8] | N/M | N/M | N/M | N/M | N/M | N/M | N/M | N/M |
| Akaberi et al., 2006[9] | N/M | N/M | N/M | N/M | N/M | N/M | N/M | N/M |
| Akaberi et al., 2008[10] | N/M | N/M | N/M | N/M | N/M | N/M | N/M | N/M |
| Al-Aradi et al., 2009[11] | General (Living: 115/774) | N/M | N/M | N/M | N/M | N/M | N/M | N/M |
| Alis et al., 2017[12] | N/M | N/M | N/M | N/M | N/M | N/M | N/M | N/M |
| Atallah et al., 2008[13] | N/M | N/M | N/M | N/M | N/M | N/M | N/M | N/M |
| Bayat et al., 2007[14] | N/M | N/M | N/M | N/M | 34 (43.6±10.9) vs. 9 (36.8± 14.3) | 34 (23.6±3.3) vs. 9 (24.9±2.9) | N/M | 34 (17.6±26.3) vs. 9 (14.8±18.8) |
| Brandenburg et al., 2002[15] | N/M | N/M | N/M | N/M | N/M | N/M | N/M | N/M |
| Brandenburg et al., 2005[16] | Lumbar (Deceased:15/67) | N/M | N/M | N/M | N/M | N/M | N/M | N/M |
| Cayco et al., 2000[17] | N/M | N/M | N/M | N/M | N/M | N/M | N/M | N/M |
| Dolgos et al., 2008[18] | N/M | Menopause (4/22) | N/M | N/M | N/M | N/M | N/M | N/M |
| Durieux et al., 2002[19] | Lumbar (Deceased:22/59)  Femoral neck (Deceased: 22/59) | N/M | N/M | N/M | N/M | N/M | N/M | N/M |
| Evenepoel et al., 2019[20] | Lumbar (Deceased:120/502)  Femoral neck (Deceased: 111/502)  Total hip (Deceased: 54/502) | DM (22/94)  Parathyroidectomy (6/68) | N/M | N/M | 111 (59.6± 11.8) vs. 115 (51±12.9) | 111 (23±3.9) vs. 115 (26.8± 4.8) | N/M | 111 (35.6±22.16) vs. 115 (33.17±26.8) |
| Evenepoel et al., 2019b[21] | Lumbar (Deceased:100/468)  Femoral neck (Deceased: 65/468) | N/M | N/M | N/M | N/M | N/M | N/M | N/M |
| Falkiewicz et al., 2009[22] | N/M | N/M | N/M | N/M | N/M | N/M | N/M | N/M |
| Ferreira et al., 2021[23] | N/M | N/M | N/M | N/M | N/M | N/M | N/M | N/M |
| Gallego et al., 2006[24] | N/M | N/M | N/M | N/M | N/M | N/M | N/M | N/M |
| Gregorini et al., 2017[25] | N/M | Menopause (17/65) | Tac (14/122)  mTOR (24/112) | N/M | N/M | 45 (23.8±3.8) vs. 179 (24.3±3.5) | N/M | N/M |
| Hsu et al., 2012[26] | N/M | N/M | N/M | N/M | 6 (56.67±9.37) vs. 31 (49.97±7.46) | 6 (20.77±2.02) vs. 31 (24.28±4.57) | 6 (39.48±21.31) vs. 31 (48.28±35.47) | N/M |
| Hsu et al., 2016[27] | N/M | N/M | N/M | N/M | 8 (58.5±9.2) vs. 33 (50.58±7.73) | 8 (21.1±2.12) vs. 33 (24.24±4.44) | 8 (50.03±34.66) vs. 33 (47.69±34.93) | N/M |
| Huang et al., 2011[28] | N/M | DM (6/13) | N/M | N/M | 48 (51±9.2) vs. 6 (49.2±3.7) | N/M | 48 (100.3±69.7) vs. 6 (133.2±57.7) | N/M |
| Gheiasi et al., 2022[29] | N/M | N/M | N/M | N/M | N/M | N/M | N/M | N/M |
| Ozel et al., 2011 [30] | N/M | N/M | Tac (3/38)  Cyc (2/6) | N/M | 5 (25.4±4.39) vs. 20 (38.15±1.12) | 5 (20.85±4.2) vs. 20 (24.75±3.85) | N/M | 5 (17.2±22.4) vs. 20 (14.3±18.2) |
| Joy et al., 2002[31] | N/M | N/M | N/M | N/M | 7 (43±12) vs. 8 (49±9) | N/M | 7 (84±42) vs. 8 (57.6±45.6) | N/M |
| Kaya et al., 2019[32] | N/M | N/M | N/M | N/M | N/M | N/M | N/M | N/M |
| Alfieri et al., 2021[33] | Lumbar (Deceased:23/32)  Femoral neck (Deceased: 25/32) | N/M | N/M | N/M | N/M | N/M | N/M | N/M |
| Wang et al., 2021[34] | N/M | Menopause (9/20) | N/M | N/M | N/M | N/M | N/M | N/M |
| Mikuls et al., 2003[35] | N/M | N/M | N/M | N/M | N/M | N/M | N/M | N/M |
| Battaglia et al., 2022[36] | N/M | DM (1/10) | N/M | N/M | 12 (59.73±6.87) vs. 33 (48.88±10.64) | 12 (23.55±2.62) vs. 33 (25.27±3.2) | 12 (71.82±85.97) vs. 33 (98.97±93.45) | 12 (40±25.85) vs. 33 (25±34.96) |
| Battaglia et al., 2023[37] | N/M | N/M | N/M | N/M | N/M | N/M | N/M | N/M |
| Mondry et al., 2001[38] | N/M | N/M | N/M | N/M | N/M | N/M | N/M | N/M |
| Pajouhi et al., 2005[39] | N/M | N/M | N/M | N/M | N/M | N/M | N/M | N/M |
| Lee et al., 2019[40] | N/M | N/M | N/M | N/M | N/M | N/M | N/M | N/M |
| Lee et al., 2014[41] | N/M | N/M | N/M | N/M | 8 (58.5±9.2) vs. 33 (50.58±7.73) | 8 (21.1±2.12) vs. 33 (24.24±4.44) | 8 (50.03±34.66) vs. 33 (47.69±34.93) | N/M |
| Opelz et al., 2011[42] | N/M | N/M | N/M | N/M | N/M | N/M | N/M | N/M |
| Park et al., 2017[43] | N/M | N/M | N/M | N/M | N/M | N/M | N/M | N/M |
| Patel et al., 2001[44] | N/M | N/M | N/M | N/M | N/M | N/M | N/M | N/M |
| Velioglu et al., 2021[45] | N/M | Menopause (6/33) | Tac (9/130)  Cyc (1/11)  mTOR (5/33) | N/M | 12 (52.7±13.2) vs. 78 (43±10.6) | 12 (25±4.9) vs. 78 (26.5±4.8) | N/M | N/M |
| Keronen et al., 2019[46] | Femoral neck (Deceased: 6/27) | N/M | N/M | N/M | N/M | N/M | N/M | N/M |
| Keronen et al., 2022[47] | N/M | N/M | N/M | N/M | N/M | N/M | N/M | N/M |
| Renau et al., 2002[48] | N/M | N/M | N/M | N/M | N/M | N/M | N/M | N/M |
| Roe et al., 2004[49] | N/M | N/M | N/M | N/M | N/M | N/M | N/M | N/M |
| Rojas et al., 2003[50] | N/M | N/M | N/M | N/M | N/M | N/M | N/M | N/M |
| Segaud et al., 2018[51] | N/M | Menopause (15/52) | N/M | N/M | N/M | N/M | N/M | N/M |
| Sezer et al., 2005[52] | N/M | N/M | N/M | N/M | 39 (31.8±10.2) vs. 19 (41.5±12.8) | 39 (21.3±2.7) vs. 19 (23.2±2.3) | N/M | N/M |
| Sikgenc et al., 2010[53] | N/M | N/M | N/M | N/M | 85 (36.3±9.9) vs. 85 (36.8±11.1) | 85 (21.4±2.5) vs. 85 (23.8±2.6) | 85 (50.7±41.3) vs. 85 (45.5±31.1) | 85 (33.1±29.3) vs. 85 (18.6±20.6) |
| Lin et al., 2023[54] | N/M | HTN (4/37)  Menopause (6/13) | Tac (7/50)  Cyc (1/14) | N/M | 8 (58.5±9.2) vs. 32 (50.81 ± 7.73) | 8 (21.1±2.12) vs. 32 (24.5±4.24) | 8 (50.03±34.66) vs. 32 (49.05±34.58) | N/M |
| Toro et al., 2003[55] | Lumbar (Deceased:18/123)  Femoral neck (Deceased: 23/123) | Menopause (20/40) | N/M | N/M | N/M | N/M | N/M | N/M |
| Ugur et al., 2001[56] | N/M | N/M | N/M | N/M | 40 (31.58±11.73) vs. 89 (30.57±9.68) | 40 (23.36±4.64) vs. 89 (24.05±2.99) | 40 (31.9±30.38) vs. 89 (27.07±36.02) | 40 (15.95±17.38) vs. 89 (17.08±17.75) |
| Unal et al., 2010[57] | N/M | N/M | N/M | N/M | 30 (38.33±11.06) vs. 10 (31.9±8.02) | 30 (24.18±3.57) vs. 10 (27.59±4.66) | N/M | N/M |
| Wang et al., 2008[58] | N/M | N/M | N/M | N/M | N/M | N/M | N/M | N/M |
| Wong et al., 2005[59] | Lumbar (Deceased:2/31)  Femoral neck (Deceased: 1/31)  Total hip (Deceased: 1/31) | N/M | N/M | N/M | N/M | N/M | N/M | N/M |
| Aleksova et al., 2017[60] | N/M | Menopause (8/38) | N/M | N/M | N/M | N/M | N/M | N/M |
| Trabulus et al., 2003[61] | N/M | N/M | N/M | N/M | N/M | N/M | N/M | N/M |
| Otaibi et al., 2021[62] | N/M | HTN (4/23) | N/M | N/M | N/M | N/M | N/M | N/M |
| Valencia et al., 2018[63] | N/M | N/M | N/M | N/M | N/M | N/M | N/M | N/M |
| Karataş et al., 2022[64] | N/M | DM (4/11)  HTN (13/45)  Menopause (11/17) | Tac (21/70)  Cyc (2/9) | N/M | N/M | 24 (27.4±8.07) vs. 15 (26.78±2.88) | N/M | 24 (42.75±36.02) vs. 15 (68.71±49.59) |
| Jørgensen et al., 2023[65] | N/M | N/M | N/M | N/M | N/M | N/M | N/M | N/M |
| Sotomayor et al., 2020[66] | N/M | DM (14/92)  HTN (51/547)  Parathyroidectomy (4/38) | Tac (13/186)  Cyc (39/309) | N/M | 65 (54±12) vs. 312 (50±13) | 65 (23.6±4.3) vs. 312 (26.6±4.2) | N/M | 65 (46±28.8) vs. 312 (37±25.3) |
| Govindarajan et al., 2011[67] | General (Living: 7/56) | N/M | N/M | N/M | N/M | N/M | N/M | N/M |
| Yavuz et al., 2022[68] | N/M | N/M | N/M | N/M | 53 (48±15.4) vs. 117 (40.2±11.3) | 53 (25.1±5.1) vs. 117 (27.2±6) | 53 (69.6±55.2) vs. 117 (84±63.6) | 53 (56.4±52.8) vs. 117 (45.6±43.2) |
| Jørgensen et al., 2021[69] | N/M | DM (10/36) | N/M | N/M | N/M | N/M | N/M | N/M |
| Sun et al., 2023[70] | Lumbar (Deceased:4/95)  Femoral neck (Deceased: 3/95) | N/M | N/M | N/M | N/M | N/M | N/M | N/M |
| ve Osteoporoz et al., 2021[71] | N/M | N/M | N/M | N/M | 24 (44.6±10.6) vs. 24 (50.4±9.98) | 24 (23.7±4.2) vs. 24 (27.3±6.08) | N/M | N/M |
| Techawathanawanna et al., 2005[72] | N/M | N/M | N/M | N/M | N/M | N/M | N/M | N/M |
| Savaj et al., 2012[73] | N/M | N/M | N/M | N/M | N/M | N/M | N/M | N/M |
| Afifi et al., 2021[74] | N/M | N/M | N/M | N/M | N/M | N/M | N/M | N/M |
| Berczi et al., 2003[75] | N/M | N/M | N/M | N/M | N/M | N/M | N/M | N/M |
| Braun et al., 2001[76] | N/M | N/M | N/M | N/M | N/M | N/M | N/M | N/M |
| Brunova et al., 2018[77] | N/M | N/M | N/M | N/M | N/M | N/M | N/M | N/M |
| Chen et al., 2016[78] | General (Living: 4/66) | N/M | N/M | N/M | N/M | N/M | N/M | N/M |
| Cruz et al., 2002[79] | N/M | N/M | N/M | N/M | N/M | N/M | N/M | N/M |
| El-Agroudy et al., 2003[80] | Forearm (Living: 2/40) | N/M | N/M | N/M | N/M | N/M | N/M | N/M |
| El-Agroudy et al., 2005[81] | Forearm (Living: 3/60) | N/M | N/M | N/M | N/M | N/M | N/M | N/M |
| Bonani et al., 2016[82] | N/M | N/M | N/M | N/M | N/M | N/M | N/M | N/M |
| Giannini et al., 2001[83] | Lumbar (Deceased:18/40)  Total hip (Deceased: 8/40) | N/M | N/M | N/M | N/M | N/M | N/M | N/M |
| Giannini et al., 2002[84] | N/M | N/M | N/M | N/M | N/M | N/M | N/M | N/M |
| Giannini et al., 2010[85] | N/M | N/M | N/M | N/M | N/M | N/M | N/M | N/M |
| Ergun et al., 2020[86] | N/M | N/M | N/M | N/M | N/M | N/M | N/M | N/M |
| Jeffery et al., 2003[87] | N/M | N/M | N/M | N/M | N/M | N/M | N/M | N/M |
| Kart-Köseoglu et al., 2003[88] | N/M | N/M | N/M | N/M | N/M | N/M | N/M | N/M |
| Kinsella et al., 2015[89] | N/M | N/M | N/M | N/M | N/M | N/M | N/M | N/M |
| Kobel et al., 2019[90] | N/M | N/M | N/M | N/M | N/M | N/M | N/M | N/M |
| Lim et al., 2009[91] | N/M | N/M | N/M | N/M | N/M | N/M | N/M | N/M |
| Małyszko et al., 2003[92] | N/M | N/M | N/M | N/M | N/M | N/M | N/M | N/M |
| Marques et al., 2019[93] | General (Living: 12/32) | N/M | N/M | N/M | N/M | N/M | N/M | N/M |
| Mazzaferro et al., 2006[94] | N/M | N/M | N/M | N/M | N/M | N/M | N/M | N/M |
| Nanmoku et al., 2019[95] | N/M | N/M | N/M | N/M | N/M | N/M | N/M | N/M |
| Naylor et al., 2014[96] | N/M | N/M | N/M | N/M | N/M | N/M | N/M | N/M |
| Naylor et al., 2014[97] | N/M | N/M | N/M | N/M | N/M | N/M | N/M | N/M |
| Nowacka-Cieciura et al., 2006[98] | N/M | N/M | N/M | N/M | N/M | N/M | N/M | N/M |
| Hameed et al., 2018[99] | N/M | DM (17/22)  HTN (20/32) | N/M | N/M | N/M | N/M | N/M | N/M |
| Rathi et al., 2015[100] | General (Living: 6/75) | N/M | N/M | N/M | N/M | N/M | N/M | N/M |
| Roberts et al., 2012[101] | General (Living: 1/73) | N/M | N/M | N/M | N/M | N/M | N/M | N/M |
| Rubello et al., 2005[102] | N/M | N/M | N/M | N/M | N/M | N/M | N/M | N/M |
| Schreiber et al., 2018[103] | N/M | N/M | N/M | Liver-Kidney (9/70) | N/M | N/M | N/M | N/M |
| Madeira et al., 2014[104] | N/M | Menopause (3/12) | N/M | N/M | 25 (43.7±13.68) vs. 63 (45±12.52) | 25 (24.2±6.76) vs. 63 (24.8±2.96) | 25 (30.33±26.73) vs. 63 (39.33±38.69) | 25 (58±56.6) vs. 63 (56.33±62.97) |
| Yu et al., 2012[105] | N/M | N/M | N/M | N/M | N/M | N/M | N/M | N/M |
| Žilinská et al., 2017[106] | N/M | N/M | N/M | N/M | N/M | N/M | N/M | N/M |
| Tsai et al., 2023[107] | N/M | N/M | N/M | N/M | N/M | N/M | N/M | N/M |
| Hori et al., 2023[108] | N/M | DM (14/60)  Parathyroidectomy (6/25) | Tac (29/120)  Cyc (60/216)  mTOR (16/49) | N/M | N/M | N/M | N/M | N/M |
| Hannarici et al., 2022[109] | N/M | N/M | N/M | N/M | N/M | N/M | N/M | N/M |
| Gupta et al., 2012[110] | N/M | DM (8/76)  Parathyroidectomy (3/12) | Tac (17/247)  Cyc (7/103)  mTOR (25/345) | N/M | 27 (56.6±9.6 vs. 178 (47.4±12.3) | 27 (23.9±5.1) vs. 178 (26.9±4.1) | 27 (81.3±99.4) vs. 178 (58.9±63.3) | N/M |
| Basir et al., 2019[111] | N/M | DM (3/9)  HTN (10/29) | N/M | N/M | N/M | N/M | N/M | N/M |
| Demir et al., 2023[112] | N/M | N/M | N/M | N/M | N/M | N/M | N/M | N/M |
| Batteux et al., 2020[113] | N/M | N/M | N/M | N/M | N/M | N/M | N/M | N/M |
| Coskun et al., 2016[114] | N/M | N/M | N/M | N/M | N/M | N/M | N/M | N/M |
| Heaf et al., 2000[115] | N/M | N/M | N/M | N/M | N/M | N/M | N/M | N/M |
| Keven et al., 2007[116] | N/M | N/M | N/M | N/M | N/M | N/M | N/M | N/M |
| Montalban et al., 2003[117] | N/M | N/M | N/M | N/M | N/M | N/M | N/M | N/M |
| Ozdem et al., 2015[118] | N/M | N/M | N/M | N/M | N/M | N/M | N/M | N/M |
| Stavroulopoulos et al., 2007[119] | N/M | N/M | N/M | N/M | N/M | N/M | N/M | N/M |
| Tutal et al., 2013[120] | N/M | N/M | Tac (13/29)  Cyc (11/26)  mTOR (18/48) | N/M | N/M | N/M | N/M | N/M |
| Matei et al., 2021[121] | N/M | N/M | N/M | N/M | N/M | N/M | N/M | N/M |
| Nouri et al., 2008[122] | N/M | N/M | N/M | N/M | N/M | N/M | N/M | N/M |
| Bonani et al., 2019[123] | N/M | N/M | N/M | N/M | N/M | N/M | N/M | N/M |
| January et al., 2020[124] | N/M | N/M | N/M | N/M | N/M | N/M | N/M | N/M |
| Prieto et al., 2009[125] | N/M | N/M | N/M | N/M | N/M | N/M | N/M | N/M |
| Tsujita et al., 2020[126] | General (Living: 21/187) | N/M | N/M | N/M | N/M | N/M | N/M | N/M |
| Hasan et al., 2020[127] | N/M | N/M | N/M | N/M | N/M | N/M | N/M | N/M |
| Alfano et al., 2018[128] | N/M | N/M | N/M | N/M | N/M | N/M | N/M | N/M |
| Huang et al., 2012[129] | N/M | DM (5/12) | N/M | N/M | N/M | N/M | N/M | N/M |
| Huang et al., 2013[130] | N/M | N/M | N/M | N/M | N/M | N/M | N/M | N/M |
| Jerman et al., 2017[131] | N/M | N/M | N/M | N/M | N/M | N/M | N/M | N/M |
| Pereira et al., 2010[132] | N/M | N/M | N/M | Pancreas-Kidney (16/57) | N/M | N/M | N/M | N/M |
| Rocha et al., 2016[133] | Lumbar (Deceased:17/48)  Femoral neck (Deceased: 19/48) | N/M | N/M | Pancreas-Kidney (36/48) | N/M | N/M | N/M | N/M |
| Smets et al., 2004[134] | N/M | N/M | N/M | N/M | N/M | N/M | N/M | N/M |
| Walder et al., 2018[135] | N/M | N/M | N/M | N/M | N/M | N/M | N/M | N/M |
| Yanishi et al., 2018[136] | N/M | N/M | N/M | N/M | N/M | N/M | N/M | N/M |

BMI: Body mass index, BMD: bone mineral density, DM: diabetes mellitus, HTN: Hypertension, Tac: Tacrolimus, Cyc: Cyclosporine, mTOR: mammalian target of rapamycin, N/M: not mentioned

1. **eFigures 1-26**

**eFigure 1.** The forest plot of the prevalence of Lumbar osteoporosis by the primary studies, and the overall estimate (95% CI).

**eFigure 2.** The forest plot of the prevalence of lumbar osteoporosis by the primary studies and the each continent, and the overall estimate (95% CI).

**eFigure 3.** The forest plot of the prevalence of lumbar osteoporosis by the primary studies and the HDI level, and the overall estimate (95% CI).

**eFigure 4.** Funnel plot of publication bias of primary studies to estimate the prevalence of lumbar osteoporosis.

**eFigure 5.** Trim and Fill analysis of the primary studies with the possibility of publication bias for the prevalence of lumbar osteoporosis.

**eFigure 6.** The forest plot of the prevalence of femoral neck osteoporosis by the primary studies, and the overall estimate (95% CI).

**eFigure 7.** The forest plot of the prevalence of femoral neck osteoporosis by the primary studies and each continent, and the overall estimate (95% CI).

**eFigure 8.** The forest plot of the prevalence of femoral neck osteoporosis by the primary studies and the HDI level, and the overall estimate (95% CI).

**eFigure 9.** Funnel plot of publication bias of primary studies to estimate the prevalence of femoral neck osteoporosis.

**eFigure 10.** Trim and Fill analysis of the primary studies with the possibility of publication bias for the prevalence of femoral neck osteoporosis.

**eFigure 11.** The forest plot of the prevalence of total hip osteoporosis by the primary studies, and the overall estimate (95% CI).

**eFigure 12.** The forest plot of the prevalence of total hip osteoporosis by the primary studies and the each continent, and the overall estimate (95% CI).

**eFigure 13.** The forest plot of the prevalence of total hip osteoporosis by the primary studies and the HDI level, and the overall estimate (95% CI).

**eFigure 14.** Funnel plot of publication bias of primary studies to estimate the prevalence of total hip osteoporosis.

**eFigure 15.** Trim and Fill analysis of the primary studies with the possibility of publication bias for the prevalence of total hip osteoporosis.

**eFigure 16.** The forest plot of the prevalence of forearm osteoporosis by the primary studies, and the overall estimate (95% CI).

**eFigure 17.** The forest plot of the prevalence of ultradistal radius osteoporosis by the primary studies, and the overall estimate (95% CI).

**
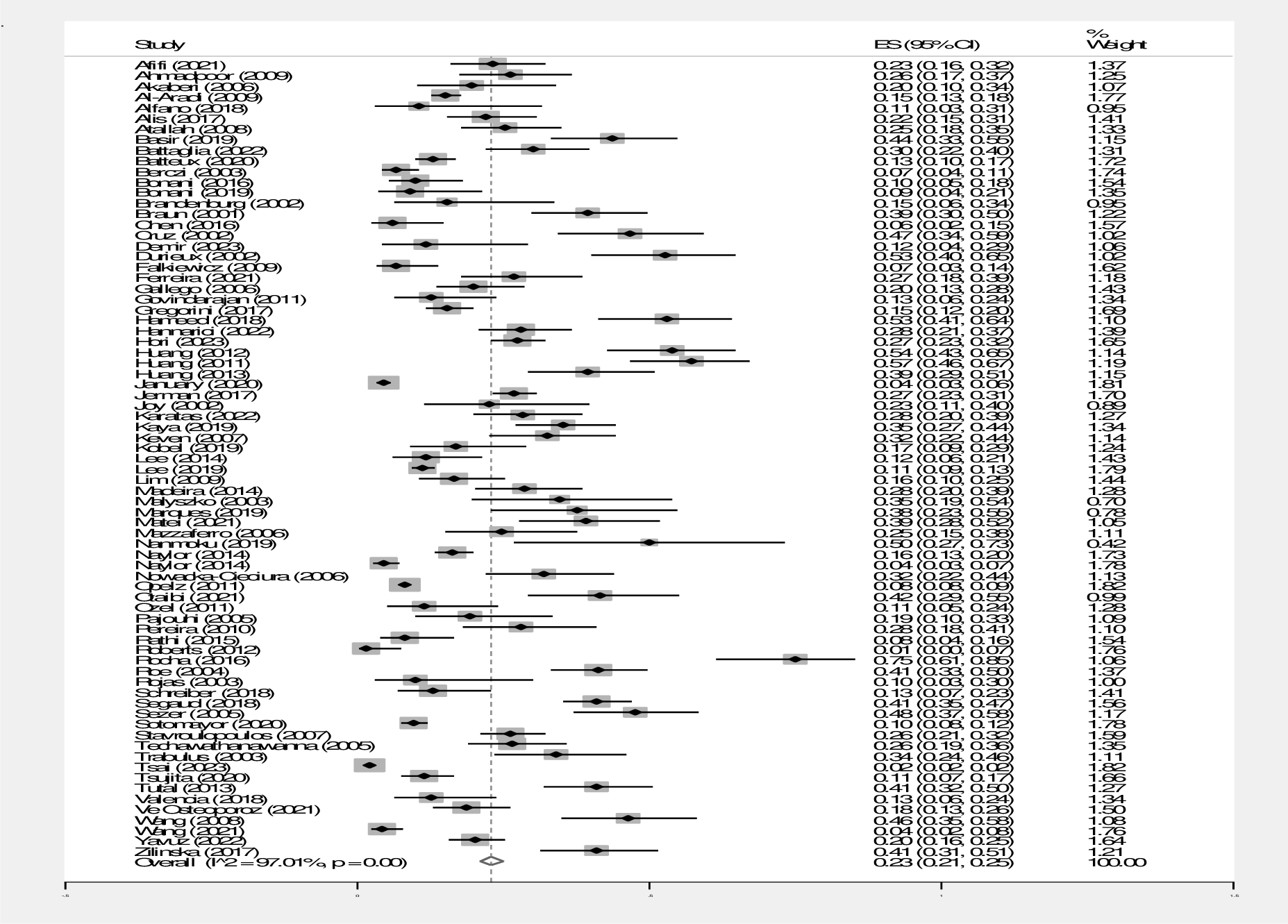
**

**eFigure 18.** The forest plot of the prevalence of general osteoporosis by the primary studies, and the overall estimate (95% CI).

**eFigure 19.** Funnel plot of publication bias of primary studies to estimate the prevalence of general osteoporosis.

**eFigure 20.** Trim and Fill analysis of the primary studies with the possibility of publication bias for the prevalence of general osteoporosis.

**eFigure 21.** The forest plot of the prevalence of osteoporosis among male kidney transplant patients by the primary studies and the overall estimate (95% CI).

**eFigure 22.** Funnel plot of publication bias of primary studies to estimate the prevalence of osteoporosis among male kidney transplant patients.

**eFigure 23.** Trim and Fill analysis of the primary studies with the possibility of publication bias for the prevalence of osteoporosis among male kidney transplant patients.

**eFigure 24.** The forest plot of the prevalence of osteoporosis among female kidney transplant patients by the primary studies and the overall estimate (95% CI).

**eFigure 25.** Funnel plot of publication bias of primary studies to estimate the prevalence of osteoporosis among female kidney transplant patients.

**eFigure 26.** Trim and Fill analysis of the primary studies with the possibility of publication bias for the prevalence of osteoporosis among female kidney transplant patients.

**eFigures 27-39**

**eFigure 27.** Prevalence of general osteoporosis among kidney transplant recipients with living donor.

**eFigure 28.** Prevalence of lumbar osteoporosis among kidney transplant recipients with deceased donor.

**eFigure 29.** Prevalence of femoral neck osteoporosis among kidney transplant recipients with deceased donor.

**eFigure 30.** Prevalence of total hip osteoporosis among kidney transplant recipients with deceased donor.

**eFigure 31.** Prevalence of forearm osteoporosis among kidney transplant recipients with living donor.

**eFigure 32.** Prevalence of osteoporosis among kidney transplant recipients with simultaneous kidney transplantation with other organs.

**eFigure 33.** Prevalence of osteoporosis among kidney transplant recipients with pre-transplant diabetes mellitus.

**eFigure 34.** Prevalence of osteoporosis among kidney transplant recipients with pre-transplant hypertension.

**eFigure 35.** Prevalence of osteoporosis among kidney transplant recipients with pre-transplant parathyroidectomy.

**eFigure 36.** Prevalence of osteoporosis among post-menopausal female kidney transplant recipients.

**eFigure 37.** Prevalence of osteoporosis among kidney transplant recipients receiving tacrolimus.

**eFigure 38.** Prevalence of osteoporosis among kidney transplant recipients receiving cyclosporine.

**eFigure 39.** Prevalence of osteoporosis among kidney transplant recipients receiving mTOR inhibitors.

**eFigures 40-43**

**eFigure 40.** Standardized mean difference for age between kidney transplant recipients with osteoporosis compared to those with normal bone mineral density.

**eFigure 41.** Standardized mean difference for body mass index between kidney transplant recipients with osteoporosis compared to those with normal bone mineral density.

**eFigure 42.** Standardized mean difference for time since transplantation between kidney transplant recipients with osteoporosis compared to those with normal bone mineral density.

**eFigure 43.** Standardized mean difference for duration of hemodialysis – pre-transplant between kidney transplant recipients with osteoporosis compared to those with normal bone mineral density.

**References**

1. Kovač D, Lindič J, Kandus A, Bren F. Quantitative ultrasound of the calcaneus and dual X-ray absorptiometry of the lumbar spine in assessment and follow-up of skeletal status in patients after kidney transplantation. Osteoporosis international. 2003;14:166-70.

2. Marcen R, Caballero C, Uriol O, Fernández A, Villafruela J, Pascual J, et al., editors. Prevalence of osteoporosis, osteopenia, and vertebral fractures in long-term renal transplant recipients. Transplantation proceedings; 2007: Elsevier.

3. Jørgensen HS, Eide IA, Hartmann A, Åsberg A, Christensen JH, Schmidt EB, et al. Plasma n-3 polyunsaturated fatty acids and bone mineral density in renal transplant recipients. Journal of Renal Nutrition. 2016;26(3):196-203.

4. Jiménez S, Marcén R, Vaamonde C, Caballero C, Fernández‐Rodríguez A, Villafruela JJ, et al. Bone fractures and lumbar mineral density after renal transplantation. A long‐term cross‐sectional study. Clinical transplantation. 2016;30(2):131-7.

5. Batteux B, Bodeau S, André C, Hurtel-Lemaire A-S, Gras-Champel V, Desailly-Henry I, et al. Association between Uremic Toxin Concentrations and Bone Mineral Density after Kidney Transplantation. Toxins. 2020;12(11):715.

6. Naga YS, Sharaki OA, Azzam EZ, Farag EMM, Zeid MMH. Relation of testosterone level and other factors with bone mineral density in male kidney transplant recipients: a cross-sectional study. BMC nephrology. 2023;24(1):271.

7. Braga Junior J, Neves R, Pinheiro MdM, Frisoli Junior A, Castro C, Szejnfeld VL, et al. Prevalence of low trauma fractures in long-term kidney transplant patients with preserved renal function. Brazilian journal of medical and biological research. 2006;39:137-47.

8. Ahmadpoor P, Reisi S, Makhdoomi K, Ghafari A, Sepehrvand N, Rahimi E, editors. Osteoporosis and related risk factors in renal transplant recipients. Transplantation proceedings; 2009: Elsevier.

9. Akaberi S, Lindergård B, Simonsen O, Nyberg G. Impact of parathyroid hormone on bone density in long-term renal transplant patients with good graft function. Transplantation. 2006;82(6):749-52.

10. Akaberi S, Simonsen O, Lindergård B, Nyberg G. Can DXA predict fractures in renal transplant patients? American Journal of Transplantation. 2008;8(12):2647-51.

11. Al-Aradi A, Phelan P, O’Kelly P, Khan A, Rahman M, Hanley A, et al. An assessment of the long-term health outcome of renal transplant recipients in Ireland. Irish journal of medical science. 2009;178:407-12.

12. Alis G, Alis M, Erturk T, Karayagiz A, Berber I, Cakir U, editors. Evaluation of bone disease in kidney transplant recipients. Transplantation Proceedings; 2017: Elsevier.

13. Atallah AM, Farag SM, Senna MK, Ghoneim MA. Musculoskeletal affections among kidney recipients: prevalence and risk predictors. Rheumatology International. 2008;28:1085-90.

14. Bayat N, Einollahi B, Pourfarzian V, Alishiri G, Nemati E, Bagheri N, et al., editors. Bone mineral density changes within 11 months of renal transplantation in Iranian patients. Transplantation proceedings; 2007: Elsevier.

15. Brandenburg VM, Ketteler M, Fassbender WJ, Heussen N, Freuding T, Floege J, et al. Development of lumbar bone mineral density in the late course after kidney transplantation. American Journal of Kidney Diseases. 2002;40(5):1066-74.

16. Brandenburg VM, Ketteler M, Heussen N, Politt D, Frank RD, Westenfeld R, et al. Lumbar bone mineral density in very long-term renal transplant recipients: impact of circulating sex hormones. Osteoporosis international. 2005;16:1611-20.

17. Cayco Av, Wysolmerski J, Simpson C, Mitnick MA, Gundberg C, Kliger A, et al. Posttransplant bone disease: evidence for a high bone resorption state. Transplantation. 2000;70(12):1722-8.

18. Dolgos S, Hartmann A, Bønsnes S, Ueland T, Isaksen GA, Godang K, et al. Determinants of bone mass in end‐stage renal failure patients at the time of kidney transplantation. Clinical transplantation. 2008;22(4):462-8.

19. Durieux S, Mercadal L, Orcel P, Dao H, Rioux C, Bernard M, et al. Bone mineral density and fracture prevalence in long-term kidney graft recipients. Transplantation. 2002;74(4):496-500.

20. Evenepoel P, Claes K, Meijers B, Laurent MR, Bammens B, Naesens M, et al. Bone mineral density, bone turnover markers, and incident fractures in de novo kidney transplant recipients. Kidney international. 2019;95(6):1461-70.

21. Evenepoel P, Claes K, Meijers B, Laurent M, Bammens B, Naesens M, et al. Poor vitamin K status is associated with low bone mineral density and increased fracture risk in end‐stage renal disease. Journal of Bone and Mineral Research. 2019;34(2):262-9.

22. Falkiewicz K, Boratyńska M, Zmonarski S, Milewicz A, Patrzałek D, Biecek P, et al., editors. Evolution of bone disease at 2 years after transplantation: a single-center study. Transplantation proceedings; 2009: Elsevier.

23. Ferreira AC, Mendes M, Silva C, Cotovio P, Aires I, Navarro D, et al. Bone densitometry versus bone histomorphometry in renal transplanted patients: a cross‐sectional study. Transplant International. 2021;34(6):1065-73.

24. Gallego R, Oliva E, Vega N, Ojeda S, Hortal L, Hernandez F, et al., editors. Steroids and bone density in patients with functioning kidney allografts. Transplantation proceedings; 2006: Elsevier.

25. Gregorini M, Sileno G, Pattonieri E, Corradetti V, Abelli M, Ticozzelli E, et al., editors. Understanding bone damage after kidney transplantation: a retrospective monocentric cross sectional analysis. Transplantation proceedings; 2017: Elsevier.

26. Hsu BG, Ho GJ, Lee CJ, Yang YC, Chen YC, Shih MH, et al. Inverse association of serum long‐acting natriuretic peptide and bone mineral density in renal transplant recipients. Clinical transplantation. 2012;26(2):E105-E10.

27. Hsu B-G, Chen Y-C, Ho G-J, Shih M-H, Chou K-C, Lin T-Y, et al., editors. Inverse association between serum osteoprotegerin and bone mineral density in renal transplant recipients. Transplantation Proceedings; 2016: Elsevier.

28. Huang W-H, Lai P-C. Age at Transplant—One of the Factors Affecting Bone Mineral Density in Kidney Recipients—A Single-Center Retrospective Study. Renal failure. 2011;33(8):776-80.

29. Gheiasi B, Hadavi M, Asadzadeh R, Taghinezhad F, Mahmodzadeh R, Mozafari A. Bone Density Reduction and Its Associated Factors in Kidney Transplant Recipients: A Cross-Sectional Study. International Journal of Organ Transplantation Medicine. 2022;13(1):5.

30. Ozel L, Ata P, Ozel M, Toros A, Kara M, Unal E, et al., editors. Risk factors for osteoporosis after renal transplantation and effect of vitamin D receptor Bsm I polymorphism. Transplantation proceedings; 2011: Elsevier.

31. Joy MS, Hollar KD, Neyhart CD, Hocan SL, Dupuis RE, Finn WF, et al. Assessment of risk factors for bone disease in renal transplant recipients. Journal of Pharmacy Technology. 2002;18(2):54-62.

32. Kaya B, Ates E, Paydas S, Sertdemir Y, Balal M, editors. Evaluation of the relationship between homocysteine, parathormone, vitamin d3, and bone mineral densitometry in recipients of kidney transplant. Transplantation Proceedings; 2019: Elsevier.

33. Alfieri C, Binda V, Malvica S, Cresseri D, Campise M, Gandolfo MT, et al. Bone effect and safety of one-year denosumab therapy in a cohort of renal transplanted patients: an observational monocentric study. Journal of Clinical Medicine. 2021;10(9):1989.

34. Wang C, Huo Y, Li X, Lin A, Hu Q, Xiong C, et al. Factors related to bone metabolism in kidney transplant recipients. Mediators of Inflammation. 2021;2021.

35. Mikuls TR, Julian BA, Bartolucci A, Saag KG. Bone mineral density changes within six months of renal transplantation. Transplantation. 2003;75(1):49-54.

36. Battaglia Y, Bellasi A, Bortoluzzi A, Tondolo F, Esposito P, Provenzano M, et al. Bone mineral density changes in long-term kidney transplant recipients: a real-life cohort study of native vitamin D supplementation. Nutrients. 2022;14(2):323.

37. Battaglia Y, Bellasi A, Esposito P, Bortoluzzi A, Rotondi S, Andreucci M, et al. The Impact of Cholecaciferol Supplementation on Bone Mineral Density in Long-Term Kidney Transplant Recipients. Biomolecules. 2023;13(4):629.

38. Mondry A, Hetzel GR, Willers R, Feldkamp J, Grabensee B. Quantitative heel ultrasound in assessment of bone structure in renal transplant recipients. American journal of kidney diseases. 2001;37(5):932-7.

39. Pajouhi M, Mahdavi-Mazdeh M, Larijani B, Soltani A, Sedaghat M, Hamidi Z, editors. Assessment of bone structure in renal transplant recipients: comparison of phalangeal qualitative ultrasound and dual x-ray absorptiometry. Transplantation proceedings; 2005: Elsevier.

40. Lee ES, Lim J-H, Cho J-H, Jung H-Y, Choi J-Y, Park S-H, et al., editors. Pretransplant osteoporosis and osteopenia are risk factors for fractures after kidney transplantation. Transplantation Proceedings; 2019: Elsevier.

41. Lee M-C, Lee C-J, Shih M-H, Ho G-J, Chen Y-C, Hsu B-G, editors. N-Terminal Pro–B-type natriuretic peptide is inversely related to bone mineral density in renal transplant recipients. Transplantation Proceedings; 2014: Elsevier.

42. Opelz G, Döhler B. Association of mismatches for HLA-DR with incidence of posttransplant hip fracture in kidney transplant recipients. Transplantation. 2011;91(1):65-9.

43. Park W, Han S, Choi B, Park C, Yang C, Kim Y-S, et al., editors. Progression of osteoporosis after kidney transplantation in patients with end-stage renal disease. Transplantation proceedings; 2017: Elsevier.

44. Patel S, Kwan JT, McCloskey E, McGee G, Thomas G, Johnson D, et al. Prevalence and causes of low bone density and fractures in kidney transplant patients. Journal of Bone and Mineral Research. 2001;16(10):1863-70.

45. Velioglu A, Kaya B, Aykent B, Ozkan B, Karapinar MS, Arikan H, et al. Low bone density, vertebral fracture and FRAX score in kidney transplant recipients: A cross-sectional cohort study. Plos one. 2021;16(4):e0251035.

46. Keronen S, Martola L, Finne P, Burton IS, Kröger H, Honkanen E. Changes in bone histomorphometry after kidney transplantation. Clinical Journal of the American Society of Nephrology. 2019;14(6):894-903.

47. Keronen S, Martola L, Finne P, Burton IS, Tong XF, Kröger H, et al. Bone volume, mineral density, and fracture risk after kidney transplantation. Plos one. 2022;17(3):e0261686.

48. Renau A, Yoldi B, Farrerons J, Sola R, Guirado L, Gich I, editors. Bone mass and mineral metabolism in kidney transplant patients. Transplantation proceedings; 2002: Orlando, FL: Grune & Stratton, 1969-.

49. Roe SD, Porter CJ, Godber IM, Hosking DJ, Cassidy MJ. Reduced bone mineral density in male renal transplant recipients: evidence for persisting hyperparathyroidism. Osteoporosis international. 2005;16:142-8.

50. Rojas E, Carlini RG, Clesca P, Arminio A, Suniaga O, De Elguezabal K, et al. The pathogenesis of osteodystrophy after renal transplantation as detected by early alterations in bone remodeling. Kidney international. 2003;63(5):1915-23.

51. Segaud N, Legroux I, Hazzan M, Noel C, Cortet B. Changes in bone mineral density after kidney transplantation: 2-year assessment of a French cohort. Osteoporosis International. 2018;29:1165-75.

52. Sezer S, Ozdemir F, Ibis A, Sayın B, Haberal M, editors. Risk factors for osteoporosis in young renal transplant recipients. Transplantation proceedings; 2005: Elsevier.

53. Sikgenc M, Paydas S, Balal M, Demir E, Kurt C, Sertdemir Y, et al., editors. Bone disease in renal transplantation and pleotropic effects of vitamin D therapy. Transplantation proceedings; 2010: Elsevier.

54. Lin W-C, Lee M-C, Chen Y-C, Hsu B-G. Inverse association of serum osteocalcin and bone mineral density in renal transplant recipients. Tzu Chi Medical Journal. 2023;35(2):165-70.

55. Toro J, Gentil M, García R, Alvárez R, Valdivia M, Roncero F, et al., editors. Osteoarticular pain and bone mineral density in renal transplantation. Transplantation proceedings; 2003: Elsevier.

56. Ugur A, Guvener N, Isklar I, Turan M, Erdal R, Haberal M. Osteoporosis after renal transplantation: single center experience. Transplantation. 2001;71(5):645-9.

57. Unal A, Kocyigit I, Sipahioglu M, Tokgoz B, Kavuncuoglu F, Oymak O, et al., editors. Loss of bone mineral density in renal transplantation recipients. Transplantation proceedings; 2010: Elsevier.

58. Wang H-H, Chang P-C, Chu S-H, Liu K-L, Lai P-C, Huang J-Y, et al., editors. Osteoporosis after kidney transplantation: preliminary report from a single center. Transplantation proceedings; 2008: Elsevier.

59. Wong H-S, Chau K-F, Wong K-M, Chan Y-H, Liu Y-L, Chan H-W, et al. Prevalence of osteoporosis in patients after renal transplantation: results from a single center. Hong Kong Journal of Nephrology. 2005;7(2):70-6.

60. Aleksova J, Wong P, Mulley WR, Choy KW, McLachlan R, Ebeling PR, et al. Serum phosphorus levels and fracture following renal transplantation. Clinical Endocrinology. 2017;87(2):141-8.

61. Trabulus S, Apaydin S, Altiparmak M, Seyahi N, Sariyar M, Serdengecti K, et al. Osteoporosis after renal transplantation. Nefrologia. 2003;23:127-30.

62. AL-Otaibi NE, Alotaibi AS, Albekairy NA, Shawaqfeh MS, Alotaibi M, Alharbi S, et al. Assessment of Risk Factors Associated with Bone and Mineral Disease Post-Renal Transplantation:“The Experiences of Two Centers”. Transplant Research and Risk Management. 2021:23-34.

63. Valencia CAR, Arango JVA, Escobar DC. Determination of vitamin D (25 [OH] D) levels in kidney transplant patients and relevance thereof, in accordance with the glomerular filtration rate. Revista Colombiana de Reumatología (English Edition). 2018;25(3):161-8.

64. Karataş A, Çanakçı E, Erdem E, Arıcı YK, Kaya M. Osteoporosis and Associated Factors in Renal Transplant Patients. Turkish journal of nephrology (Online). 2022;31(1):66-73.

65. Jørgensen HS, Claes K, Smout D, Naesens M, Kuypers D, D’Haese P, et al. Associations of Changes in Bone Turnover Markers with Change in Bone Mineral Density in Kidney Transplant Patients. Clinical Journal of the American Society of Nephrology. 2023:10.2215.

66. Sotomayor CG, Benjamens S, Gomes-Neto AW, Pol RA, Groothof D, te Velde-Keyzer CA, et al. Bone mineral density and aortic calcification: evidence for a bone-vascular axis after kidney transplantation. Transplantation. 2021;105(1):231-9.

67. Govindarajan S, Khandelwal N, Sakhuja V, Jha V. Bone mineral density in patients with end-stage renal disease and its evolution after kidney transplantation. Indian Journal of Nephrology. 2011;21(2):85-9.

68. Gogas Yavuz D, Aydin K, Apaydin T, Velioglu A, Mert M, Pekkolay Z, et al. Clinical predictors of incipient vertebral fractures and bone mineral density in kidney transplant patients. European Spine Journal. 2022;31(9):2423-30.

69. Jørgensen HS, Behets G, Bammens B, Claes K, Meijers B, Naesens M, et al. Patterns of renal osteodystrophy 1 year after kidney transplantation. Nephrology Dialysis Transplantation. 2021;36(11):2130-9.

70. Sun L, Huang Z, Fei S, Ni B, Wang Z, Chen H, et al. Vascular calcification progression and its association with mineral and bone disorder in kidney transplant recipients. Renal Failure. 2023;45(2):2276382.

71. ve Osteoporoz BNHO, Eden AİEİT, Parametre A. Anthropometric parameter that best predicts the relationship between obesity and osteoporosis in kidney transplant recipients. Turk J Osteoporos. 2021;27:8-13.

72. Techawathanawanna N, Avihingsanon Y, Praditpornsilpa K, Kingpetch K, Suwanwalaikorn S, Kanjanabuch T, et al. The prevalence and risk factors of osteoporosis in Thai renal-transplant patients. JOURNAL-MEDICAL ASSOCIATION OF THAILAND. 2005;88:S103.

73. Savaj S, Ghods FJ. Vitamin D, parathyroid hormone, and bone mineral density status in kidney transplant recipients. Iranian Journal of Kidney Diseases. 2012;6(4):295.

74. Afifi MK, Kenawy AS, El Demellawy HH, Azouz AA, Al-Otaibi T, Gheith O, et al. Optimization of osteoporosis and osteopenia management among renal transplant recipients. Future Journal of Pharmaceutical Sciences. 2021;7:1-7.

75. Berczi C, Asztalos L, Kincses Z, Balogh A, Lőcsey L, Balazs G, et al. Comparison of calcium and alfacalcidol supplement in the prevention of osteopenia after kidney transplantation. Osteoporosis international. 2003;14:412-7.

76. Braun W, Protiva D, editors. Emerging profiles in 105 recipients of renal allografts functioning for 20 to 35 years: the" watershed" effect. Transplantation proceedings; 2001.

77. Brunova J, Kratochvilova S, Stepankova J. Osteoporosis therapy with denosumab in organ transplant recipients. Frontiers in Endocrinology. 2018;9:162.

78. Chen Z, Sun J, Haarhaus M, Barany P, Wennberg L, Ripsweden J, et al. Bone mineral density of extremities is associated with coronary calcification and biopsy-verified vascular calcification in living-donor renal transplant recipients. Journal of Bone and Mineral Metabolism. 2017;35:536-43.

79. Cruz DN, Brickel HM, Wysolmerski JJ, Gundberg CG, Simpson CA, Kliger AS, et al. Treatment of osteoporosis and osteopenia in long‐term renal transplant patients with alendronate. American Journal of Transplantation. 2002;2(1):62-7.

80. El-Agroudy AE, El-Husseini AA, El-Sayed M, Ghoneim MA. Preventing bone loss in renal transplant recipients with vitamin D. Journal of the American Society of Nephrology. 2003;14(11):2975-9.

81. El-Agroudy AE, El-Husseini AA, El-Sayed M, Mohsen T, Ghoneim MA. A prospective randomized study for prevention of postrenal transplantation bone loss. Kidney international. 2005;67(5):2039-45.

82. Bonani M, Frey D, Brockmann J, Fehr T, Mueller TF, Saleh L, et al. Effect of twice-yearly denosumab on prevention of bone mineral density loss in de novo kidney transplant recipients: a randomized controlled trial. American Journal of Transplantation. 2016;16(6):1882-91.

83. Giannini S, D'Angelo A, Carraro G, Nobile M, Rigotti P, Bonfante L, et al. Alendronate prevents further bone loss in renal transplant recipients. Journal of Bone and Mineral Research. 2001;16(11):2111-7.

84. Giannini S, D'Angelo A, Nobile M, Carraro G, Rigotti P, Silva‐Netto F, et al. The effects of vitamin D receptor polymorphism on secondary hyperparathyroidism and bone density after renal transplantation. Journal of Bone and Mineral Research. 2002;17(10):1768-73.

85. Giannini S, Sella S, Silva Netto F, Cattelan C, Dalle Carbonare L, Lazzarin R, et al. Persistent secondary hyperparathyroidism and vertebral fractures in kidney transplantation: Role of calcium‐sensing receptor polymorphisms and vitamin D deficiency. Journal of bone and mineral Research. 2010;25(4):841-8.

86. ERGÜN İ. Changes in Bone Mineral Density after Kidney Transplantation. Duzce Medical Journal. 2020;22(1):41-5.

87. Jeffery JR, Leslie WD, Karpinski ME, Nickerson PW, Rush DN. Prevalence and treatment of decreased bone density in renal transplant recipients: a randomized prospective trial of calcitriol versus alendronate. Transplantation. 2003;76(10):1498-502.

88. Kart-Köseoglu H, Yücel A, Isıklar I, Türker I, Akcalı Z, Haberal M. Joint pain and arthritis in renal transplant recipients and correlation with cyclosporine therapy. Rheumatology international. 2003;23:159-62.

89. Kinsella S, Murphy K, Breen M, O’Neill S, McLaughlin P, Coyle J, et al. Comparison of single CT scan assessment of bone mineral density, vascular calcification and fat mass with standard clinical measurements in renal transplant subjects: the ABC HeART study. BMC nephrology. 2015;16:1-11.

90. Kobel C, Frey D, Graf N, Wüthrich RP, Bonani M. Follow-up of bone mineral density changes in de novo kidney transplant recipients treated with two doses of the receptor activator of nuclear factor κB ligand inhibitor denosumab. Kidney and Blood Pressure Research. 2019;44(5):1285-93.

91. Lim WH, Coates PS, Russ GR, Coates PTH. Hyperparathyroidism and vitamin D deficiency predispose to bone loss in renal transplant recipients. Transplantation. 2009;88(5):678-83.

92. Małyszko J, Wołczyński S, Małyszko J, Konstantynowicz J, Kaczmarski M, Myśliwiec M, editors. Correlations of new markers of bone formation and resorption in kidney transplant recipients. Transplantation proceedings; 2003: Elsevier.

93. Marques IDB, Araújo MJCLN, Graciolli FG, Dos Reis LM, Pereira RMR, Alvarenga JC, et al. A randomized trial of zoledronic acid to prevent bone loss in the first year after kidney transplantation. Journal of the American Society of Nephrology. 2019;30(2):355-65.

94. Mazzaferro S, Diacinti D, Proietti E, Barresi G, Baldinelli M, Pisani D, et al. Morphometric X-ray absorptiometry in the assessment of vertebral fractures in renal transplant patients. Nephrology Dialysis Transplantation. 2006;21(2):466-71.

95. Nanmoku K, Shinzato T, Kubo T, Shimizu T, Yagisawa T. Effects of denosumab on hypercalcemia and bone mineral density loss in kidney transplant recipients. Clinical nephrology. 2019;92(1):1.

96. Naylor KL, Leslie WD, Hodsman AB, Rush DN, Garg AX. FRAX predicts fracture risk in kidney transplant recipients. Transplantation. 2014;97(9):940-5.

97. Naylor KL, Garg AX, Hodsman AB, Rush DN, Leslie WD. Long-term changes in bone mineral density in kidney transplant recipients. Transplantation. 2014;98(12):1279-85.

98. Nowacka-Cieciura E, Cieciura T, Bączkowska T, Kozińska-Przybył O, Tronina O, Chudziński W, et al., editors. Bisphosphonates are effective prophylactic of early bone loss after renal transplantation. Transplantation proceedings; 2006: Elsevier.

99. Hameed EO, Sinjari HY. Prevalence and risk factors of osteoporosis in kidney transplant recipients: dual-Energy x-ray absorptiometry scan study. Medical Journal of Babylon. 2018;15(4):267-70.

100. Rathi M, Kumar D, Bhadada SK, Khandelwal N, Kohli HS, Jha V, et al. Sequential changes in bone biochemical parameters and bone mineral density after renal transplant. Saudi Journal of Kidney Diseases and Transplantation. 2015;26(4):671-7.

101. Roberts L, Ramsaroop K, Seemungal T. Survival Outcomes in Renal Transplantation in Trinidad and Tobago SORTTT Study. West indian medical journal. 2012;61(4).

102. Rubello D, Giannini S, D’Angelo A, Nobile M, Carraio G, Rigotti P, et al. Secondary hyperparathyroidism is associated with vitamin D receptor polymorphism and bone density after renal transplantation. Biomedicine & pharmacotherapy. 2005;59(7):402-7.

103. Schreiber PW, Bischoff-Ferrari HA, Boggian K, Bonani M, van Delden C, Enriquez N, et al. Bone metabolism dynamics in the early post-transplant period following kidney and liver transplantation. PLoS One. 2018;13(1):e0191167.

104. Madeira M, Zen MS, Lacativa PGS, Torres CH, Lázaro APP, Gonçalves RT, et al. Decreased bone mineral density in patients submitted to kidney transplantation is related to age, body mass index, time on dialysis, and hyperparathyroidism. Advances in Endocrinology. 2014;2014.

105. Yu RW, Faull RJ, Coates PTH, Coates PS. Calcium supplements lower bone resorption after renal transplant. Clinical transplantation. 2012;26(2):292-9.

106. Žilinská Z, Dedinská I, Breza J, Laca Ľ. Effect of Paricalcitol on Bone Density After Kidney Transplantation. Iranian Journal of Kidney Diseases. 2017;11(6).

107. Tsai H-L, Lin T-C, Lin N-C, Yang H-H, Chang J-W. Risk Factors for Fractures in Renal Transplantation: A Population-Based Cohort Study. American Journal of Nephrology. 2023;54(11-12):498-507.

108. Kondoa MHHTC, Matsuokac Y, Nishihirad MTM, Takedaa KUA, Maruyamae KMS. Coexistence of low muscle mass and osteoporosis as a predictor of fragility fractures in long-term kidney transplant recipients. Am J Nephrol. 2023;54:489-97.

109. Hannarici Z, Uçler R, Yıldız S, Soyoral YU, Alay M. Evaluation of Clinical and Laboratory Factors Affecting Bone Mineral Density Measurements in Patients with Kidney Transplant. Journal of Clinical Densitometry. 2022;25(3):343-8.

110. Gupta AK, Huang M, Prasad G. Determinants of bone mineral density in stable kidney transplant recipients. Journal of nephrology. 2012;25(3):373-83.

111. Basir H, Altunoren O, Erken E, Kilinc M, Sarisik FN, Isiktas S, et al. Relationship Between Osteoporosis and Serum Sclerostin Levels in Kidney Transplant Recipients. Experimental and clinical transplantation: official journal of the Middle East Society for Organ Transplantation. 2019.

112. Demir C, Dursun AD, Sarıyıldız GT, Arslan Aİ. Serum irisin levels and osteoporosis in patients with advanced chronic kidney disease and renal transplant recipients. International Urology and Nephrology. 2023;55(7):1821-8.

113. Batteux B, Gras-Champel V, Lando M, Brazier F, Mentaverri R, Desailly-Henry I, et al. Early steroid withdrawal has a positive effect on bone in kidney transplant recipients: a propensity score study with inverse probability-of-treatment weighting. Therapeutic advances in musculoskeletal disease. 2020;12:1759720X20953357.

114. Coskun Y, Paydas S, Balal M, Soyupak S, Kara E, editors. Bone disease and serum fibroblast growth factor-23 levels in renal transplant recipients. Transplantation Proceedings; 2016: Elsevier.

115. Heaf J, Tvedegaard E, Kanstrup IL, Fogh‐Andersen N. Bone loss after renal transplantation: role of hyperparathyroidism, acidosis, cyclosporine and systemic disease. Clinical transplantation. 2000;14(5):457-63.

116. Keven K, Ozturk R, Sengul S, Kutlay S, Ergun I, Erturk S, et al. Renal tubular acidosis after kidney transplantation—incidence, risk factors and clinical implications. Nephrology Dialysis Transplantation. 2007;22(3):906-10.

117. Montalban C, De Francisco AL, Mariñoso ML, Zubimendi JA, Unzueta MG, Amado JA, et al. Bone disease in long-term adult kidney transplant patients with normal renal function. Kidney International. 2003;63:S129-S32.

118. Ozdem S, Yılmaz VT, Ozdem SS, Donmez L, Cetinkaya R, Suleymanlar G, et al. Is Klotho F352V polymorphism the missing piece of the bone loss puzzle in renal transplant recipients? Pharmacology. 2015;95(5-6):271-8.

119. Stavroulopoulos A, Cassidy M, Porter C, Hosking D, Roe S. Vitamin D status in renal transplant recipients. American Journal of Transplantation. 2007;7(11):2546-52.

120. Tutal E, Uyar M, Colak T, Bal Z, Demirci B, Bozkurt T, et al., editors. Low graft function and ongoing hyperparathyroidism are closely related to post-transplantation osteoporosis. Transplantation proceedings; 2013: Elsevier.

121. Matei A, Bilha SC, Constantinescu D, Pavel-Tanasa M, Cianga P, Covic A, et al. Body composition, adipokines, FGF23-Klotho and bone in kidney transplantation: Is there a link? Journal of nephrology. 2021:1-12.

122. Nouri-Majalan N, Sanadgol H, Rahimian M, Soleimani H. Bone mineral density in kidney transplant recipients and patients on hemodialysis: a comparison with healthy individuals. Iranian journal of kidney diseases. 2008;2(3):154-9.

123. Bonani M, Frey D, Graf N, Wüthrich RP. Effect of denosumab on trabecular bone score in de novo kidney transplant recipients. Nephrology Dialysis Transplantation. 2019;34(10):1773-80.

124. January SE, Progar K, Nesselhauf NM, Hagopian JC, Malone AF. Choice of Acid Suppressant Therapy and Long‐Term Graft Outcomes After Kidney Transplantation. Pharmacotherapy: The Journal of Human Pharmacology and Drug Therapy. 2020;40(11):1082-8.

125. Prieto FT, Blanco GB, García MN, Chaves VC, Garciajiménez R, Palomo PP, et al., editors. Calcimimetics and bone mineral density in renal transplant patients with persistent secondary hyperparathyroidism. Transplantation proceedings; 2009: Elsevier.

126. Tsujita M, Doi Y, Obi Y, Hamano T, Tomosugi T, Futamura K, et al. Cholecalciferol supplementation attenuates bone loss in incident kidney transplant recipients: a prespecified secondary endpoint analysis of a randomized controlled trial. Journal of Bone and Mineral Research. 2020;37(2):303-11.

127. Hasan AA, Al-Temimi HMA. Evaluation the Risk Factors that are Associated with Osteoporosis in Post Kidney Transplantation in a Sample of Iraqi Patients. Iraqi Journal of Pharmaceutical Sciences (P-ISSN 1683-3597 E-ISSN 2521-3512). 2020;29(2):1-7.

128. Alfano G, Mori G, Fontana F, Dolci G, Baisi A, Ligabue G, et al. Clinical outcome of kidney transplantation in HIV-infected recipients: a retrospective study. International journal of STD & AIDS. 2018;29(13):1305-15.

129. Huang W-H, Lee S-Y, Weng C-H, Lai P-C. Use of alendronate sodium (Fosamax) to ameliorate osteoporosis in renal transplant patients: a case-control study. PLoS One. 2012;7(11):e48481.

130. Huang W-H, Yu M-C, Huang J-Y, Lai P-C. Impact of hepatitis C virus infection on bone mineral density in renal transplant recipients. Plos one. 2013;8(5):e63263.

131. Jerman A, Lindič J, Škoberne A, Borštnar Š, Bergoč MM, Godnov U, et al. Prevalence and risk factors for nonvertebral bone fractures in kidney transplant recipients–a single-center retrospective analysis. Clinical nephrology. 2017;88(7):101.

132. Pereira S, Pedroso S, Martins L, Santos P, Almeida M, Freitas C, et al., editors. Bone mineral density after simultaneous kidney–pancreas transplantation: four years follow-up of 57 recipients. Transplantation proceedings; 2010: Elsevier.

133. Rocha A, Martins LS, Malheiro J, Dores J, Santos C, Henriques C. Changes in bone mineral density following long-term simultaneous pancreas-kidney transplantation. Journal of bone and mineral metabolism. 2016;34:209-15.

134. Smets YF, De Fijter JW, Ringers J, Lemkes HH, Hamdy NA. Long-term follow-up study on bone mineral density and fractures after simultaneous pancreas-kidney transplantation. Kidney international. 2004;66(5):2070-6.

135. Walder A, Müller M, Dahdal S, Sidler D, Devetzis V, Leichtle AB, et al. The effect of a previous created distal arteriovenous-fistula on radial bone DXA measurements in prevalent renal transplant recipients. Plos one. 2018;13(7):e0200708.

136. Yanishi M, Kinoshita H, Tsukaguchi H, Kimura Y, Koito Y, Sugi M, et al., editors. Factors related to osteosarcopenia in kidney transplant recipients. Transplantation Proceedings; 2018: Elsevier.
